# A Review of Mechanoluminescence in Inorganic Solids: Compounds, Mechanisms, Models and Applications

**DOI:** 10.3390/ma11040484

**Published:** 2018-03-23

**Authors:** Ang Feng, Philippe F. Smet

**Affiliations:** 1 LumiLab, Department of Solid State Sciences, Ghent University, Krijgslaan 281-S1, 9000 Ghent, Belgium; Ang.Feng@UGent.be; 2 Center for Nano- and Biophotonics (NB Photonics), Ghent University, 9000 Ghent, Belgium

**Keywords:** mechanoluminescence, elastic deformation, stress distribution sensing, mechanoluminescence mechanism, piezoelectricity, defects, persistent luminescence

## Abstract

Mechanoluminescence (ML) is the non-thermal emission of light as a response to mechanical stimuli on a solid material. While this phenomenon has been observed for a long time when breaking certain materials, it is now being extensively explored, especially since the discovery of non-destructive ML upon elastic deformation. A great number of materials have already been identified as mechanoluminescent, but novel ones with colour tunability and improved sensitivity are still urgently needed. The physical origin of the phenomenon, which mainly involves the release of trapped carriers at defects with the help of stress, still remains unclear. This in turn hinders a deeper research, either theoretically or application oriented. In this review paper, we have tabulated the known ML compounds according to their structure prototypes based on the connectivity of anion polyhedra, highlighting structural features, such as framework distortion, layered structure, elastic anisotropy and microstructures, which are very relevant to the ML process. We then review the various proposed mechanisms and corresponding mathematical models. We comment on their contribution to a clearer understanding of the ML phenomenon and on the derived guidelines for improving properties of ML phosphors. Proven and potential applications of ML in various fields, such as stress field sensing, light sources, and sensing electric (magnetic) fields, are summarized. Finally, we point out the challenges and future directions in this active and emerging field of luminescence research.

## 1. Introduction

Luminescence is the emission of cold light due to different kinds of excitation sources, in contrast to the black body radiation as encountered in, e.g., incandescent lamps. Broadly speaking, luminescence as a response to mechanical stimuli is termed mechanoluminescence. This phenomenon was first recorded by Francis Bacon in his book *The Advancement of Learning* (1605) as “it is not the property of fire alone to give light...loaf-sugar in scraping or breaking” [1]. Yet, it must have been known centuries earlier because sugar canes were already imported by Europe since the 12th century [2].

Triboluminescence was historically used as a synonym for ML [2], but nowadays it refers to luminescence due to the contact of two dissimilar materials [3]. The process often involves a triboelectric field, a chemical reaction or just the generation of heat in the contact area. In a narrower sense, ML refers to luminescence induced by elastic deformation (elasticoluminescence), plastic deformation (plasticoluminescence), or fracture (fractoluminescence) depending on the magnitude of stress. Fractoluminescence was reported in as many as a thousand compounds and can be found in 36% of inorganic, and even 50% of all crystalline materials, according to an estimate in the late 1990s [4]. It is mainly attributed to the breaking of chemical bonds, which charges ambient nitrogen gas or/and luminescent dopants [5,6,7], and has found applications in sensors for damage monitoring [8]. The study of its mechanism is complicated by the difficulty in assessing the influence of crystal size, the mechanical load and the surrounding gasses, etc.

**Figure 1:**
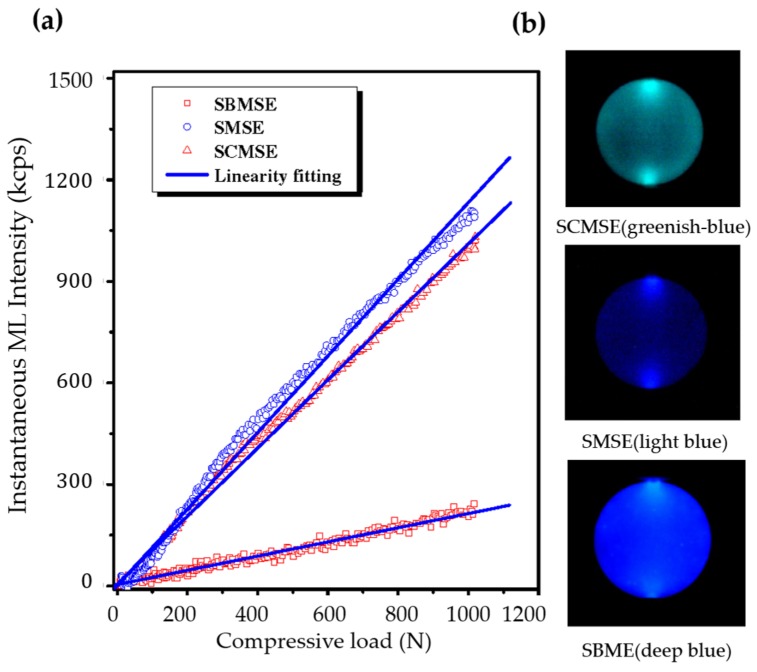
ML intensity-load relationship (**a**) and optical ML images (**b**) of SrAMgSi${}_{2}$O${}_{7}$:Eu${}^{2+}$ for A = Ca, Sr and Ba, noted as SCMSE, SMSE and SBMSE, respectively. Reproduced from Ref [11] Copyright (2009) The Japan Society of Applied Physics.

The discovery of elasticoluminescence in particular phosphors, such as SrAl${}_{2}$O${}_{4}$:Eu${}^{2+}$, Dy${}^{3+}$ [9] and ZnS:Mn${}^{2+}$ [10] in the late 1990s, has sparkled enthusiasm among scientists to research ML upon non-destructive elastic deformation. As an example in Figure 1a,b, the instantaneous ML intensity induced by elastic deformation of SrAMgSi${}_{2}$O${}_{7}$:Eu${}^{2+}$ (A = Ca, Sr, Ba) is proportional to the stress load applied at a fixed rate [11]. Based on the linearity between ML intensity and stress, the stress distribution of a composite under compression [12], tensile load [13], torsion [14,15], ultrasound radiation [16] or vibration due to gas flow [17] has been estimated by this ML-based method. The stress field and wake in the vicinity of a crack [18,19], and its propagation [20,21] can also be visualized in an in situ manner thanks to ML phosphors. Recent reports showed that ML materials are able to emit light upon applying electric [13] or magnetic [22] fields when they are properly coupled with piezoelectric crystals (magnetic alloys).

Despite the great potential in different areas, novel ML phosphors have been mainly discovered in a trial-and-error process, evidenced by only a few tens of intense ML phosphors and their limited ranges of colours. Arguably, this results from the limited understanding of the mechanism behind the ML phenomenon. The emission of light in these ML phosphors under dynamic loading is due to a transition of electrons from the excited state to the ground state of dopants (transition metals, lanthanides, etc.). Intuitively, these electrons are generated upon deformation or are released from traps in phosphors where charge carriers were trapped during the photo-excitation process [23]. Indeed, the average mechanical energy gained by an atom during elastic deformation ($10^{-6}-10^{-5}\;$eV is in principle not sufficient to directly excite it into emitting visible light, which requires a few eVs [7] (*According to the infinitesimal theory, the strain energy density u (J/m*${}^{3}$*) can be estimated as* $u=\frac{\sigma^{2}}{2E}$ *in which Young’s modulus* $E=\frac{9KG}{3K+G}$*, with K bulk modulus and G shear modulus. As an example, SrAl*${}_{2}$*O*${}_{4}$*:*1%*Eu*${}^{2+}$ *has theoretical density of 3493 kg/m*${}^{3}$*,* K=44.7 *GPa,* G=27.7 *GPa [24], which leads to* $u=1.82\times 10^{4}\;$*J/m*${}^{3}$*, i.e.,* $4.0\times 10^{-6}\;$*eV/atom, in a typical ML experiment where 50 MPa can be expected (Appendix A)*). Carriers are often stored in traps created by dopants/co-dopants or by cation/anion vacancies in phosphors. They can also escape from these traps by thermal energy, resulting in the delayed emission of light, which is termed persistent luminescence and is now reasonably well understood (see Refs [25,26] for a review), although the nature of (de)trapping process at defects remains unclear [27]. Alternatively, charge carriers can also be released upon mechanical load and subsequently recombine with luminescent centres, eventually yielding ML. Consequently, ML and persistent luminescence often take place simultaneously, which complicates the study of the detrapping mechanism.

It is still rather mysterious how carriers are facilitated to be released during elastic or plastic deformation of a ML phosphor. The piezoelectricity of host compounds was assumed to provide an internal electric field which helps carriers to overcome barriers, in order to escape from defects [28]. Piezoelectricity induced by breaking inversion symmetry via doping was also argued to be responsible for ML in CaNb${}_{2}$O${}_{6}$:Pr${}^{3+}$ and Ca${}_{3}$Nb${}_{2}$O${}_{8}$:Pr${}^{3+}$, which share centrosymmetric hosts [29]. However, neither persistent luminescence nor piezoelectricity in a certain phosphor guarantees the occurrence of ML. How the geometric configuration of defects, together with their energy levels, changes under deformation still remains unclear. In addition, strain-induced changes of microstructures, such as twin boundaries and domain walls [30], impose complexity to the problem. For these reasons, it is no wonder that substantial advancement in understanding ML phosphors came at a slow pace during the past two decades. In addition to a review on triboluminescence in 1977 [7], there are a few reviews available in the field of ML research at present. Some are too concise to take a deep dive into the structure clarification and mechanism study [31,32]. The other focuses only on lanthanide ML, which also include organic molecules, and does not relate ML to the research of persistent luminescence [33]. Therefore, we provide in this review an extensive overview of the field of ML. ML materials are first tabulated and possible trends of the crystal structure and microstructures are pointed out. A summary of proposed mechanisms is given and it is discussed how theoretical models improve our understanding of ML. Next we provide an overview of demonstrated and potential applications of ML materials. Finally, we highlight some of the remaining challenges and identify specific future research tracks in this particularly active area of phosphor research and applications.

## 2. Basic Concepts

ML is essentially related to detrapping of carriers in phosphors under dynamic loading, but the relatively small number of ML phosphors available makes it far from straightforward to better understand its mechanism and to distil guidelines for discovering new ML phosphors. However, it is instructive to assess first the roles of the host symmetry, the defects and the types of external mechanical load in ML. Finally, it is important to gain insight in how ML phosphors are commonly measured and evaluated.

### 2.1. Symmetry and Tensor Properties in Crystals

The full crystallographic symmetry of a crystal can be described by one of the 230 space groups. Modulation vectors can be added to describe incommensurate modulated structures [34]. Among these symmetry elements, point group is the most important because it is common to all of its physical properties (Neumann’s Principle [35]). Under an external influence, the induced phenomenon can be obtained through a physical property, which can often be described by a tensor (*A tensor is a multilinear transformation which takes r vectors (r is also the rank of the tensor) as input and produces a number [36]. In physics, a tensor and its associated vectors are often physical properties (electric field, stress, etc.). Multilinearity ensures the independency of the chosen way of description and a consistent relationship between the components expressed in different coordinates*). As the symmetry of a tensor is related to the medium and its intrinsic symmetry, the independent components and their relations can be gained by various methods, such as matrix method [37], direct inspection [38] and group theory [39].

For a better understanding of the ML phenomenon, both the crystallographic properties of the host and the effect induced by stress are required. The electric polarization per unit cell of insulating crystalline solid is the centre of charge of Wannier functions of the occupied bands [40]. In non-centrosymmetric compounds (excluding those with point group 432), electric polarization can be generated by applying a stress and the effect is termed piezoelectricity [41]. Permanent electric polarization can be present in polar crystals and leads to pyroelectricity, an effect where charges are generated by uniform changes of temperature [41]. Ferroelectricity is obtained when spontaneous polarization can be switched by an external field (temperature, electric field, etc.) [42]. Furthermore, polarization can also be generated by gradients of strain, which is called flexoelectricity for which the coefficients form a fourth rank tensor [43]. In addition to electric polarization, spontaneous strain can exist in crystals as well. In parallel, if the spontaneous strain can be switched by an applied external stress, the phenomenon is called ferroelasticity [44]. Another effect of stress on crystals is to change the refractive index (i.e., the photoelastic effect [45]), for which the tensor is called the piezo-optical coefficient. The symmetry of stress itself and the point group of crystals determine the relations of tensor components, as shown in Appendix B for piezoelectric coefficients and piezo-optical coefficients.

### 2.2. Dynamic Deformation and Its Consequences

The central role of dynamic finite strain (stress) in ML calls for a careful consideration of the definition and their consequences. Strain, a physical observable quantifying deformation of continuum matter, is now often described by a function of metric tensors $g_{i}^{0}$ (initial configuration) and $g_{i}$ (current configuration) in co-moving coordinates. For example, Green-Love strain in classical finite deformation theory is defined as $\varepsilon_{ij}=(g_{ij}-g_{ij}^{0})/2$ [46]. All information on deformation is contained in the transformation $F_{i}^{j}$ that changes $g_{i}^{0}$ to $g_{i}$, i.e., $g_{i}=F_{i}^{j}g_{i}^{0}$. Here,

(1) $$F_{i}^{j}=\delta_{i}^{j}+u^{j}{|}_{i}$$

where $\delta_{i}^{j}$ is Kronecker delta, and $u^{j}{|}_{i}\equiv \frac{\partial u^{i}}{\partial x^{j}}+\Gamma_{il}^{j}u^{l}$ is the covariant derivative of displacement field in which $\Gamma_{il}^{j}$ is the Christoffel symbols of the second kind [47]. Based on the inner product of base vector, Green-Love strain fails to describe the local rotation correctly, which is very important for dynamic load, especially for large deformation. Chen, Z.-D. [48] suggested $F_{i}^{j}$ be decomposed into a symmetric tensor $S_{i}^{j}$ and an orthogonal tensor $R_{i}^{j}$ (*Another decomposition scheme, i.e., the polar decomposition, was formulated as:* F=RU=VR*, in which R, U and V are the orthogonal rotation tensor, the right stretch tensor and the left stretch tensor, respectively [49]. It is clear that such decomposition results in two unique stretch tensors and a temporal order of rotation and extension (compression). In such a sense, it does not place a very solid foundation of mechanics*):

(2) $$F_{i}^{j}=S_{i}^{j}+R_{i}^{j}$$

with

$$S_{i}^{j}=\frac{1}{2}\left(u^{i}{|}_{j}+{u^{i}{|}_{j}}^{T}\right)-\left(1-\cos\Theta \right)L_{l}^{i}L_{j}^{l},\;R_{i}^{j}=\delta_{i}^{j}+\sin\Theta \cdot L_{j}^{i}+\left(1-\cos\Theta \right)L_{l}^{i}L_{j}^{l}$$

in which,

$$\Theta =\arcsin\left(\frac{1}{2}\sqrt{{\left(u^{1}{{|}_{2}-u^{2}|}_{1}\right)}^{2}+{\left(u^{2}{{|}_{3}-u^{3}|}_{2}\right)}^{2}+{\left(u^{1}{{|}_{3}-u^{3}|}_{1}\right)}^{2}}\right),\;L_{j}^{i}=\frac{1}{2\sin\Theta }\left(u^{i}{|}_{j}-{u^{i}{|}_{j}}^{T}\right).$$

Here, T means transpose. Clearly, $S_{i}^{j}$ is an extension of Cauchy’s strain definition to finite deformation in an intrinsic co-moving system, while $R_{i}^{j}$ corresponds to finite rotation in Euler parameters, with $L_{j}^{i}$ being the rotation axis and Θ the rotation angle (*The Cauchy strain is defined in fixed Cartesian coordinates (*$X^{j}$*) by the displacement field* $u^{i}$ *as* $\varepsilon_{j}^{i}=\frac{\partial u^{i}}{\partial X^{j}}\left(i,j=1,2,3\right)$*. In 3d space, a central rotation around the unit vector* k *by an angle θ can be expressed in a concise form* $R=\left(\cos\left(\theta \right)\right)I+\left(\sin\left(\theta \right)\right){\left[k\right]}_{\times }+\left(1-\cos\left(\theta \right)\right)\left(k⊗k\right)$*, where* ${\left[k\right]}_{\times }$ *is the cross product of* k*,* ⊗ *the tensor product and **I** the identity matrix.*). In such sense, the strain can be simply defined as

(3) $$\begin{array}{cc} \varepsilon_{i}^{j} & =F_{i}^{j}-\delta_{i}^{j}\\ & =S_{i}^{j}+\left(R_{i}^{j}-\delta_{i}^{j}\right) \end{array}$$

Here, $R_{i}^{j}$ and $\left(S_{i}^{j}-\delta_{i}^{j}\right)$ correspond also to the longitudinal wave (P-wave, P for primary) and transverse wave (S-wave, S for secondary) in the theory of elastic wave [50]. A non-zero Θ means irreversible strain residual even though the macrodeformation is “reversible” (*The reverse transformation of* $F_{j}^{i}$ *is denoted as* ${\tilde{F}}_{j}^{i}$*, satisfying* $F_{l}^{i}{\tilde{F}}_{j}^{l}=\delta_{j}^{i}$*. If* ${\tilde{F}}_{j}^{i}={\tilde{S}}_{j}^{i}+{\tilde{R}}_{j}^{i}$*, then a non-zero Θ means* $\left(S_{l}^{i}+R_{l}^{i}\right)\left({\tilde{S}}_{j}^{l}+{\tilde{R}}_{j}^{l}\right)=S_{l}^{i}{\tilde{S}}_{j}^{l}+S_{l}^{i}{\tilde{R}}_{j}^{l}+R_{l}^{i}{\tilde{S}}_{j}^{l}+R_{l}^{i}{\tilde{R}}_{j}^{l}=\delta_{j}^{i}$*. Suppose the rotation is reversible,* $R_{l}^{i}{\tilde{R}}_{j}^{l}=\delta_{j}^{i}$*, then we have* ${\tilde{R}}_{j}^{i}={R_{j}^{i}}^{T}$ *and* $S_{l}^{i}S_{j}^{l}+S_{l}^{i}{\tilde{R}}_{j}^{l}+R_{l}^{i}{\tilde{S}}_{j}^{l}=0$*. In principle,* $S_{l}^{i}{\tilde{R}}_{j}^{l}+R_{l}^{i}{\tilde{S}}_{j}^{l}\neq 0$ *and therefore* $S_{j}^{i}+{\tilde{S}}_{i}^{j}\neq 0$*, which shows irreversibility*), which may result in plasticity and fatigue [51], an effect that is often neglected.

Stress is normally viewed as the response of strain due to external forces, and is often defined as Cauchy stress, which in Cartesian coordinates reads,

(4) $$\sigma_{j}^{i}=\lambda \left(\frac{\partial u^{l}}{\partial X^{l}}\right)\delta_{j}^{i}+2\mu \frac{\partial u^{i}}{\partial X^{j}}$$

where λ and μ are Lamé constants. Here, it is assumed that the continuum media react to external strain spontaneously. In the elastic limit, this relation can be expressed as Hooke’s Law:

(5) $$\sigma_{j}^{i}=E_{jl}^{ik}\cdot \varepsilon_{k}^{l}$$

in which $E_{jl}^{ik}$ is the elastic stiffness tensor with rank 4 and has only 36 independent components for static loads. In dynamic loads, the requirement of conservation of momentum and angular momentum leads to,

(6) $$\sigma_{j}^{i}-{\sigma_{j}^{i}}^{T}=\frac{\partial }{\partial t}\left[\left(\rho \frac{\partial U^{i}}{\partial t}\right)g_{il}^{0}\left(F_{j}^{l}-\delta_{j}^{l}\right)\right]$$

This relation means that stress is always asymmetric under dynamic loads and is symmetric in static loads [51].

**Figure 2:**
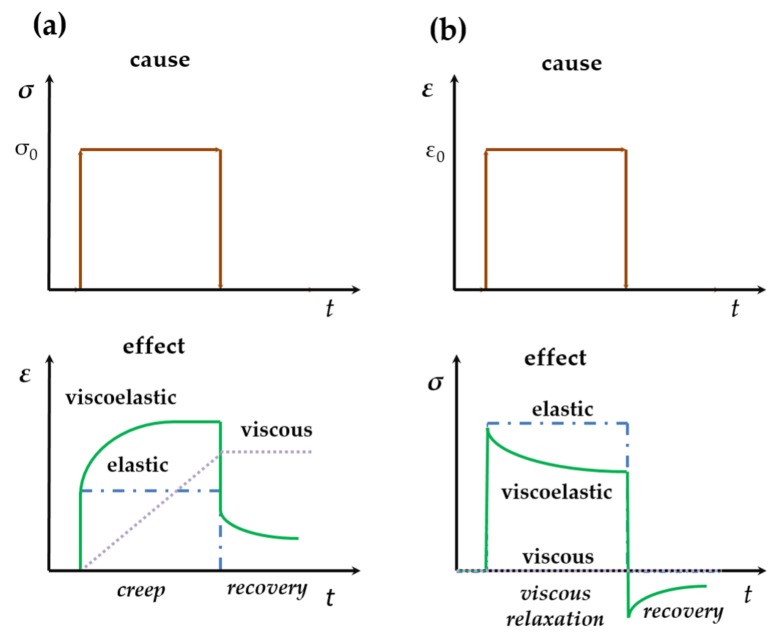
The strain evolution under step stress load (**a**) and stress evolution under step strain load (**b**) for elastic, viscous and viscoelastic materials. Reproduced from Ref [56], with permission from Cambridge University Press.

In reality, stress may also depend on the history of loading, where elastic stiffness changes with time to a certain value. Following the theory of linear viscoelasticity, stress can then be calculated [52],

(7) $$\sigma \left(t\right)=\int_{0}^{t}E\left(t-\tau \right)\frac{\partial \varepsilon \left(\tau \right)}{\partial \tau }d\tau$$

thanks to the Boltzmann superposition principle [53]. According to Chen’s decomposition, the constitutive relation is now,

(8) $$\sigma_{j}^{i}\left(t_{0},t\right)=E_{jk}^{im}\int_{t_{0}}^{t}s_{m}^{k}\left(\tau \right)d\tau +\left[1-\cos\left(\int_{t_{0}}^{t}\dot{\Theta_{j}^{i}}\left(\tau \right)d\tau \right)\right]L_{m}^{l}L_{l}^{k}E_{jk}^{im}$$

where the second term corresponds to viscous stress which is here associated with local rotation [54]. Typical phenomena of viscoelasticity are stress relaxation (a step constant strain results in decreasing stress), creep (a step constant stress results in increasing strain), and hysteresis (a phase lag in stress-strain relation). The response to stress (strain) of elastic, viscous and viscoelastic materials are drawn schematically in Figure 2, for the purpose of comparison.

Viscoelasticity has also been observed in other physical properties, such as piezoelectricity, ferromagnetism, dielectricity, which account for the non-linearity of response to external fields and are argued to be related to domain structures [55]. Similarly, the mechanical response of the ML material itself has to be taken into account when investigating the mechanism of ML.

### 2.3. Measurement and Performance of ML Materials

**Figure 3:**
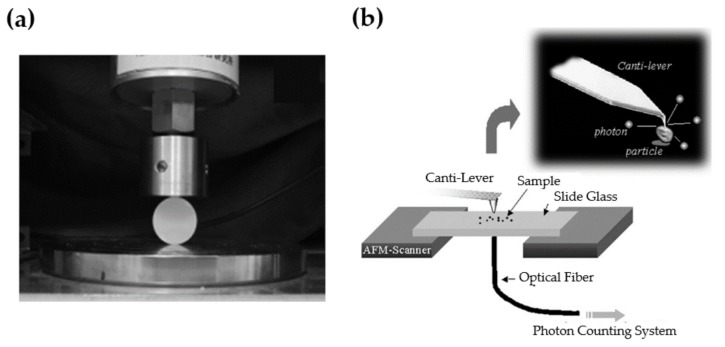
A setup for testing the ML response under compressive load (**a**); and a setup for testing ML for a single microparticle (**b**). Reproduced from Ref [57], Copyright (2012), with permission from Elsevier and Ref [58] with permission of The Royal Society of Chemistry, respectively.

A setup capable of deforming materials can be utilized to measure ML, combined with additional devices to collect the emission of light. A typical apparatus introduced by Xu et al. is shown in Figure 3a [57], where the force (or displacement) can be read from load actuators and the instantaneous ML intensity can be recorded by photomultiplier tubes (PMT), photodiodes, high speed cameras or portable spectrometers. It is also possible to detect ML at the micro-scale (Figure 3b) by attaching a photo-counting system to an atomic force microscope [58]. This method is helpful to gain insights into the relationship between the strain and ML intensity, although the signal is inevitably quite weak. Standardized measurement procedures are needed to compare ML results from different phosphors or setups, for example using calibrated detectors that quantify absolute ML energy [59].

A good ML phosphor often exhibits high ML intensities and excellent linearity between ML intensity and mechanical load, in addition to other merits of phosphors in lighting applications. The ML intensity carries a twofold meaning, namely the instantaneous intensity and the accumulated intensity. The former refers to the emitted power of photons detected by a camera or PMT subtracted by the instantaneous intensity of persistent luminescence free of mechanical load. The latter means the integration of instantaneous intensity from time when the load begins to any time of interest. The instantaneous intensity is often proportional to the mechanical power applied into ML phosphors and the slope between these quantities indicates the sensitivity to loads. The load range in which the instantaneous ML intensity is linear to mechanical loads, or range of linearity in brief, is a good indicator of ML phosphors for the capability of sensing stress. Of course, the storage capacity (for example SrAl${}_{2}$O${}_{4}$:Eu${}^{2+}$, Dy${}^{3+}$ has a capacity of 1.6 × 10${}^{17}$ photons/g [60]) and the initial fraction of the traps filled by photon charging determine the intensity of the trap controlled ML. Saturation, which makes ML independent of trap filling, can be obtained by increasing the duration or the intensity of the excitation during the charging step.

## 3. Mechanoluminescent Compounds

### 3.1. Known ML Compounds

In the early stage of ML research, more than a thousand compounds were reported to be luminescent due to fracture, i.e., fractoluminescence. Colored alkali halides and ZnS:Mn${}^{2+}$ single crystals also exhibited ML under elastic or plastic deformations [7]. Elastico- or plastico-luminescence in phosphors gained interest only since the late 1990s. Hence, several criteria are set to screen the latter ML compounds. Crystals that show fractoluminescence are excluded here because its nature is associated with charge separation near crystal facets, providing only limited information on the physics of luminescence under deformation. Compounds showing ML due to impact are compiled only when the impact is of a sufficiently low velocity such that fracture is negligible. Persistent luminescence, if any, and depths of the relevant traps are noted when available in literature. Before ML experiments, phosphors are often charged by light of appropriate wavelength. In Table 1 , these compounds are listed in several groups based on their structural types (or their derivative structures), for the purpose of revealing common structural features and their impact on ML. In this regard, the preparation methods and post treatment were not accounted here though they are important for the final properties of phosphors. Interested readers are referred to other sources, such as Refs [61,62].

### 3.2. Crystal Structures and Their Relation to ML

#### 3.2.1. Rock Salt and Wurtzite-Related Compounds

In rock salt compounds, which are built up with layers of edge-sharing octahedra, colour centres are often created by irradiation of X-rays, γ-rays or by treatment with metal vapor. Dislocations in these single crystals are prone to slip as the critical stress is only about 1 MPa [199]. Actually, ML was found in coloured alkali halides early since the 1930s, but non-irradiated alkali halides usually do not provide light during deformation except for fracture [7]. ML takes place even at a very low stress levels (see Table 1) and its intensity is linear to the stress within the elastic limit but is only proportional to mechanical power during plastic deformation [200]. In successive load cycles, a memory effect can be observed: ML only appears after new plastic deformation takes place [201].

**Figure 4:**
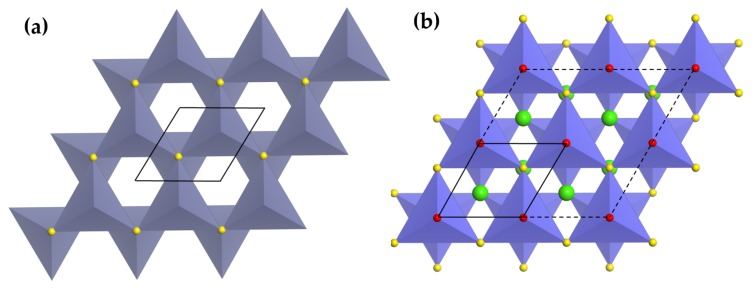
Crystal structure of ZnS (**a**) along [00$\bar{1}$] and crystal structure of CaZnOS (**b**) as a 2 × 2 × 1 supercell (dashed line) along [001]. Unit cells are outlined by solid lines. (S${}^{2-}$: yellow, Ca${}^{2+}$: green, O${}^{2-}$: red.)

An analogue of rock salt structures, wurzite-ZnS is a hexagonal close packing of layers connected by [ZnS${}_{4}$] tetrahedra sharing common anions (Figure 4a). The layers are repeated in a …ABABA… fashion along (001) planes. The absence of inversion centres results in piezoelectricity [202]. ML has been found in ZnS doped with Cu${}^{+}$, or Mn${}^{2+}$, which was argued to arise from the interaction between piezoelectricity and shallow acceptor and donor levels [203]. It is still unclear how these phosphors recover ML intensity to initial levels in successive load cycles (self-recovery) [10,108,121,204], although strong retrapping of electrons in the conduction band was proposed for such phenomenon [205].

CaZnOS can be classified as a wurtzite-related compound in terms of the packing of tetrahedra layers. These layers consist of [ZnS${}_{3}$O] tetrahedra sharing common S in the basal plane and at z = **c**/2, while Ca${}^{2+}$ ions are inserted into channels between these layers (Figure 4b)). All Zn-O bonds run parallel and point to the -**c** direction, making CaZnOS a polar compound characterized by a large piezoelectric constant ($d_{33}$ = 38 pm/V) [206]. CaZnOS was found to be ML when doped with Cu${}^{+}$, Mn${}^{2+}$, or lanthanides (Er${}^{3+}$, Sm${}^{3+}$), though there is no self-recovery of ML intensity as in the case of ZnS. For both ZnS and CaZnOS, persistent luminescence was not observed for various dopants except for Cu${}^{+}$, indicating that also deeper defects are possibly responsible for ML. Interestingly, BaZnOS contains layers of vertex-linked [ZnO${}_{2}$S${}_{2}$] tetrahedra and adopts non-centrosymmetric space group Cmcm [207]. It gives red ML (634 nm) when doped with Mn${}^{2+}$, adding an example of ML in non-piezoelectric host [141,142].

#### 3.2.2. Tridymites

**Figure 5:**
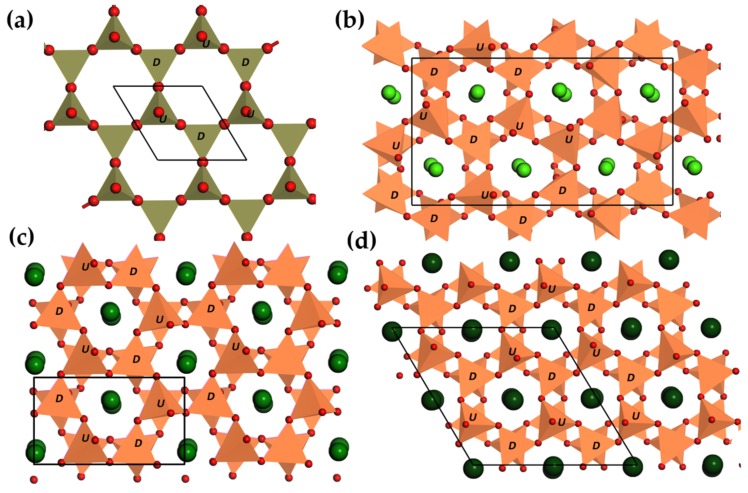
Structure of tridymite (**a**) along [001], crystal structure of CaAl${}_{2}$O${}_{4}$ unit cell (**b**) along [010], crystal structure of SrAl${}_{2}$O${}_{4}$ supercell (1×2×2) (**c**) along [100], and crystal structure of BaAl${}_{2}$O${}_{4}$ supercell (1×2×1) (**d**) along [001]. Unit cells are outlined by black lines, and the direction of tetrahedra was marked as U(up) and D (down). (Ca${}^{2+}$: green, Sr${}^{2+}$: deep green, Ba${}^{2+}$: heavy green, O${}^{2-}$: red.)

Tridymite (Figure 5a), a high temperature polymorph of silica, is an excellent example to illustrate the bonding topology of framework structures, which are typical for silicates and aluminates. Vertex sharing [SiO${}_{4}$] tetrahedra are often connected to form ring-like nets in planes, such that framework structures are formed when the third dimension is subject to further connections. The topology of ring-like structures is characterized by the sequence of upward (U) or downward (D) pointing of tetrahedra, e.g., UDUDUD for tridymite (Figure 5a). It is also possible to replace Si${}^{4+}$ ions by ions of lower oxidation states, on condition that charge neutrality is achieved by suitable cations. These cations are stuffed in cavities, resulting in stuffed tridymite structures. Accordingly, different topologies of tetrahedron arrangements or/and structural distortions lead to a large number of supercells and structures of low symmetry [208].

AAl${}_{2}$O${}_{4}$ (A = Ca, Sr, Ba) adopt the stuffed tridymite structure, but display structural differences in tetrahedra connectivity and site symmetry of cations. The three structures contain similar motifs consisting of rings with corner-sharing [AlO${}_{4}$] tetrahedra, but the sequence of tetrahedra corners are slightly different. In CaAl${}_{2}$O${}_{4}$, the tetrahedra are heavily tilted and rotated, exhibiting a new tetrahedron sequence, DDUDUU, in addition to the common UDUDUD sequence (Figure 5b). Small Ca${}^{2+}$ ions can be accommodated in three different coordination environments: two sites are coordinated by six O atoms from AlO${}_{4}$ tetrahedra, while the other site is ninefold coordinated [209]. On the contrary, both SrAl${}_{2}$O${}_{4}$ and BaAl${}_{2}$O${}_{4}$ adopt a UDUDUD tetrahedra corner sequence (Figure 5c,d). BaAl${}_{2}$O${}_{4}$ features higher symmetry (P6${}_{3}$), in which Ba${}^{2+}$ are ninefold coordinated. Sr${}^{2+}$ ions are so small for the large cavities in the undistorted framework that they occupy two nonequivalent sites to have irregular polyhedra [210]. [AlO${}_{4}$] tetrahedra are tilted with respect to the (001) plane in addition to a cooperative rotation along the [001] direction [211]. It is noteworthy that SrAl${}_{2}$O${}_{4}$ lattice is highly anisotropic since the thermal expansion coefficients along **a** and **b** axis are an order of magnitude larger than the one along **c** axis [212]. When doped with Eu${}^{2+}$ or Ce${}^{3+}$, SrAl${}_{2}$O${}_{4}$ is among the most studied ML compounds mainly due to its high brightness of ML. The distortion of tetrahedra in Sr${}_{1-x}$Ba${}_{x}$Al${}_{2}$O${}_{4}$ (x= 0–1.0) can be lifted by either raising temperature or by incorporating bigger cation (thus by increasing *x*) [211], and a transformation from monoclinic to hexagonal structure takes place around $x_{c}=$ 0.31–0.43. Accidentally, the ML intensity of Sr${}_{1-x}$Ba${}_{x}$Al${}_{2}$O${}_{4}$:Eu${}^{2+}$, Eu${}^{3+}$ decreases with increasing *x* and reaches zero around $x_{c}=0.4$, the critical point [98]. Thus, the ML intensity seems to be related to the distortion of the tetrahedra framework since piezoelectricity and persistent luminescence are present throughout the whole composition range. Furthermore, twins, boundaries or planar defects are often found in low temperature form of these compounds mainly due to phase transitions [213,214]. Interestingly, when pulse loads *P* (<20 MPa) were applied to CaAl${}_{2}$O${}_{4}$: Eu${}^{2+}$, the instantaneous ML intensity *I* follows $I\propto \exp\left(-1/\sqrt{P}\right)$ , which was argued to originate from electroluminescence upon piezoelectric field [93]. This different behaviour can provide insights for the mechanism of ML in this group of phosphors.

Zn${}_{2}$GeO${}_{4}$ is a derivative structure of tridymite by replacing all [SiO${}_{4}$] by [ZnO${}_{4}$] and [GeO${}_{4}$] in a ratio of 2:1. These tetrahedra are no longer arranged parallel to the **a**-**b** plane, but tilted in such a way that every tetrahedron gets one edge along [001] [215]. Persistent luminescence being weak, Zn${}_{2}$GeO${}_{4}$:Si${}^{4+}$, Mn${}^{2+}$ shows green ML under compressive loads [100].

#### 3.2.3. Anorthite and Melilites

**Figure 6:**
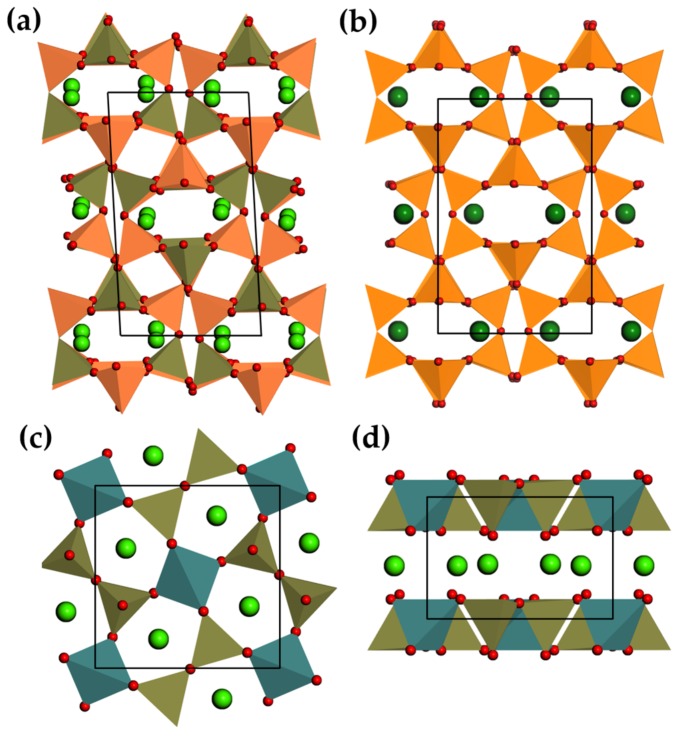
The structure of CaAl${}_{2}$Si${}_{2}$O${}_{8}$ (**a**) along [001], the structure of SrAl${}_{2}$Si${}_{2}$O${}_{8}$ (**b**) along [001], and the structure of melilite Ca${}_{2}$MgSi${}_{2}$O${}_{7}$ along [001] (**c**), and along [100] (**d**). (Ca${}^{2+}$: green, Sr${}^{2+}$: deep green, O${}^{2-}$: red, [SiO${}_{4}$]: earth, [AlO${}_{4}$]: brown, [MgO${}_{4}$]: deep cayan, [(Al, Si)O${}_{4}$]: orange.)

Anorthite is an end member of plagioclase feldspar: NaAlSi${}_{3}$O${}_{8}$ (albite)-CaAl${}_{2}$Si${}_{2}$O${}_{8}$ (anorthite). It exhibits complexity in anion connectivity and cation orders, while having structure modulations or phase transitions in albite-anorthite solid solutions or Ca${}_{1-x}$Sr${}_{x}$Al${}_{2}$Si${}_{2}$O${}_{8}$ (0 < *x* < 1) series. For anorthite as shown in Figure 6a, a framework structure composed of four-member rings is formed by [SiO${}_{4}$] and [AlO${}_{4}$] tetrahedra in an ordered manner, but disorders of tetrahedra may also be present since spontaneous strains are often found, leading to ferroelasticity. Chemical substitution reveals the influence of cation size on the crystal chemistry of feldspar. Ca${}_{1-x}$Sr${}_{x}$Al${}_{2}$Si${}_{2}$O${}_{8}$ transforms to monoclinic structure at $x_{c}=$ 0.86 as the content of Sr increases [216], which also shows characteristics of ferroelasticity. The order parameters, i.e., internal strains $\varepsilon_{12}$ and $\varepsilon_{23}$, disappear at the compositional transition point $x_{c}=$ 0.86, indicating that SrAl${}_{2}$Si${}_{2}$O${}_{8}$ is fully disordered in the [AlO${}_{4}$] and [SiO${}_{4}$] tetrahedra [216] (Figure 6b). It is surprising to find that ML disappears at $x_{c}$ [170], which suggests that internal strains play an important role for ML in this anorthite series.

The crystal structure of SrMg${}_{2}$(PO${}_{4}$)${}_{2}$ is still unavailable but its zinc analogue, α-SrZn${}_{2}$(PO${}_{4}$)${}_{2}$, is constructed from a three-dimensional network which is isotypic to BaAl${}_{2}$Si${}_{2}$O${}_{8}$ [217]. For this reason, it was grouped into anorthite related compounds based on the similar ionic size of Mg${}^{2+}$ and Zn${}^{2+}$ ($r_{Mg^{2+}}$ = 0.83 Å, $r_{Zn^{2+}}$ = 0.74 Å for CN = 4 [218]), and their tendency to be tetrahedrally coordinated by O atoms [219].

Melilite phosphors have the general formula X${}_{2}$T${}^{1}$T${}_{2}^{2}$O${}_{7}$, with a large cation X = Na, Ca-Ba, Pb or lanthanides, while T${}^{1}$ = Mg, Zn, Al, Si, and T${}^{2}$ = Si, Ge, Al, B are often coordinated by oxygen ions. They crystallize in a tetragonal structure with space group P$\bar{4}$2${}_{1}$m. Two [T${}^{2}$O${}_{4}$] tetrahedra are corner-linked to form [T${}_{2}^{2}$O${}_{7}$] dimers, which again are linked to four surrounding [T${}^{1}$O${}_{4}$] tetrahedra via bridging oxygen, thus forming tetrahedral sheets parallel to (001). The irregular pentagonal arrangements create large channels along the **c**-axis, in which X cations are located halfway between successive sheets [220]. The structure of Ca${}_{2}$MgSi${}_{2}$O${}_{7}$ is shown in Figure 6c,d as an example. The strain due to the misfit between X cations and the tetrahedra sheet can be relaxed by rotation or twisting of tetrahedra together with possible atomic displacements of bridging O atoms [221,222], which is often thought as the structural reason for phase transitions in melilite compounds. Furthermore, particular ordering patterns of low-coordinated cations are directly linked to incommensurate structures [223,224]. Interestingly, the internal strains are so sensitive to cation size that alloying the cation site could even tune the modulation wavelength and phase transition temperature [225]. Meanwhile, incorporating Al into both T${}^{1}$ (Mg) and T${}^{2}$ sites (Si) tends to destroy the modulation structure (e.g., modulation structure is absent in Ca${}_{2}$Al${}_{2}$SiO${}_{7}$) and results in Al-Si order at the T${}^{2}$ site [226] and cation vacancies as a consequence of avoiding Al-O-Al bonds between [AlO${}_{4}$] tetrahedra (“Loewenstein rule” [227]) [228].

It is clear from Table 1 that ML can be found in Ce${}^{3+}$ or Eu${}^{2+}$ doped anorthite and melilite, which also show persistent luminescence. Due to their unique structures, elasticity anisotropy can be expected from melilite and anorthite compounds. This was indeed confirmed by determination of the elastic stiffness constants from first principle calculation and experimental data [229,230,231,232]. Elastic anisotropy may help to reduce symmetry and hence to release trapped electrons when compounds are subjected to strain (especially shear strain), causing ML.

#### 3.2.4. Perovskite Related Compounds

**Figure 7:**
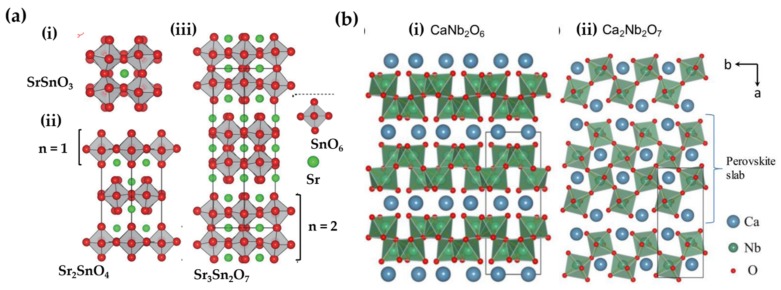
Crystal structure of Ruddlesden-Popper structures Sr${}_{n+1}$Sn${}_{n}$O${}_{3n+1}$ (**a**) viewed along [010], where **(i)** n = *∞*, **(ii)** n = 1, and **(iii)** n = 2; crystal structure of calcium niobates mCaO·Nb${}_{2}$O${}_{5}$ (**b**) for **(i)** m = 1, and **(ii)** m = 2. Reproduced from Refs [29,183] with the permission of AIP Publishing and Copyright (2016) American Chemical Society, respectively.

Simple as it may seem, ABO${}_{3}$ perovskites consist of corner-sharing [BO${}_{6}$] octahedra adopting a primitive cubic unit cell, with the cations A stuffed in 12-coordinated interstices (Figure 7a–i). Site A can accommodate two kinds of cations as long as their sum of oxidation states is 6. The effect is two-fold: on one hand, the size requirement between site A and site B can result in twisted or distorted structures, while on the other hand cation ordering is also possible. The tunability of perovskite compounds, which can be achieved by altering the crystal structure and defects configurations by means of varying chemical composition or synthesis procedures, has made perovskites successful in many applications, such as piezoelectric transducers [233], superconductors [234], catalysts [235], and phosphors [236]. Perovskite Ba${}_{1-x}$Ca${}_{x}$TiO${}_{3}$ (0.25 < *x* < 0.9) is composed of the tetragonal ferroelectric phase Ba${}_{0.77}$Ca${}_{0.23}$TiO${}_{3}$ and the orthorhombic normal dielectric Ba${}_{0.1}$Ca${}_{0.9}$TiO${}_{3}$ [175]. Coupled with ferroelectric domains in Ba-rich phases, the polarization of Ca-rich phases was argued to induce ML in this system since large internal electric fields can be triggered under strain [175,237]. The structure of LiNbO${}_{3}$, can be described by a displacement of hexagonally closed-packed oxygens, which makes it a ferroelectric crystal [238] ($d_{15}$ = 69.2 pC/N). It is also a transformation from its high temperature structure, which is isomorphous to perovskite [239].

Sr${}_{n+1}$Sn${}_{n}$O${}_{3n+1}$ (n = 1, 2, *∞*) are termed Ruddlesden-Popper structures (Figure 7(aii,iii)), but they can be regarded as perovskite-rocksalt intergrowth structures, described by SrO(SrSnO${}_{3}$)${}_{n}$, in which a rock-salt layer SrO grows with every n layers of perovskite SrSnO${}_{3}$ [240]. To minimize the mismatch (i.e., strain) between SnO${}_{2}$ and Sr${}_{2}$O${}_{2}$ layers, the [SnO${}_{6}$] octahedra are tilted cooperatively in the case of n = 1 or 2 [240]. The sensitivity to cation size indicates that these compounds may adopt at high temperatures crystal structures with high symmetry to lower internal strains. As a comparison, BaSn${}_{2}$O${}_{4}$ (I$\frac{4}{m}$mm) differs from its strontium counterpart by the absence of octahedral distortions. Tilted [SnO${}_{6}$] octahedra in CaSn${}_{2}$O${}_{4}$ (Pbam) form one dimensional chains by sharing edges [182]. Sm${}^{3+}$ doped Sr${}_{n+1}$Sn${}_{n}$O${}_{3n+1}$ (n = 1, 2, *∞*) are persistent luminescent materials for which the number of traps per volume (trap concentration) reduces as n increases from 1 to infinity [183]. The trap concentration and ML intensity were improved by alloying the cation site in Sr${}_{2}$SnO${}_{4}$ [186], or alloying [SnO${}_{6}$] tetrahedra with Si or Ge in the case of Sr${}_{3}$Sn${}_{2}$O${}_{7}$:Sm${}^{3+}$ [187]. Sr${}_{n+1}$Sn${}_{n}$O${}_{3n+1}$ (n = 1, 2, *∞*): Sm${}^{3+}$ shows ML, although they are not intrinsically piezoelectric. It is still possible that weak piezoelectricity is induced by defects at the microscale. Another member in Ruddlesden-Popper structures is Ca${}_{3}$Ti${}_{2}$O${}_{7}$, which shows ML when doped with Pr${}^{3+}$ [189].

Recently, Zhang et al. revealed ML in perovskite related niobates mCaO·Nb${}_{2}$O${}_{5}$ (m = 1–3) doped with Pr${}^{3+}$, in which CaO layers are inserted into layers of Nb-O polyhedral (Figure 7(bi)) or perovskite slabs (Figure 7(bii)) [29]. Although CaNb${}_{2}$O${}_{6}$ and Ca${}_{3}$Nb${}_{2}$O${}_{8}$ are centrosymmetric, they showed piezoelectricity due to the anisotropy or cation vacancies of the hosts. Defects are created in these compounds, but lead to persistent luminescence only in Ca${}_{2}$Nb${}_{2}$O${}_{7}$. The absence of the blue component of ML spectra with respect to photoluminescence emission spectra was argued to originate from a direct tunneling of electrons from intrinsic defect centres to Pr${}^{3+}$.

#### 3.2.5. MSi${}_{2}$O${}_{2}$N${}_{2}$: Eu${}^{2+}$ (M = Ba, Sr, Ca) and Other Compounds

**Figure 8:**
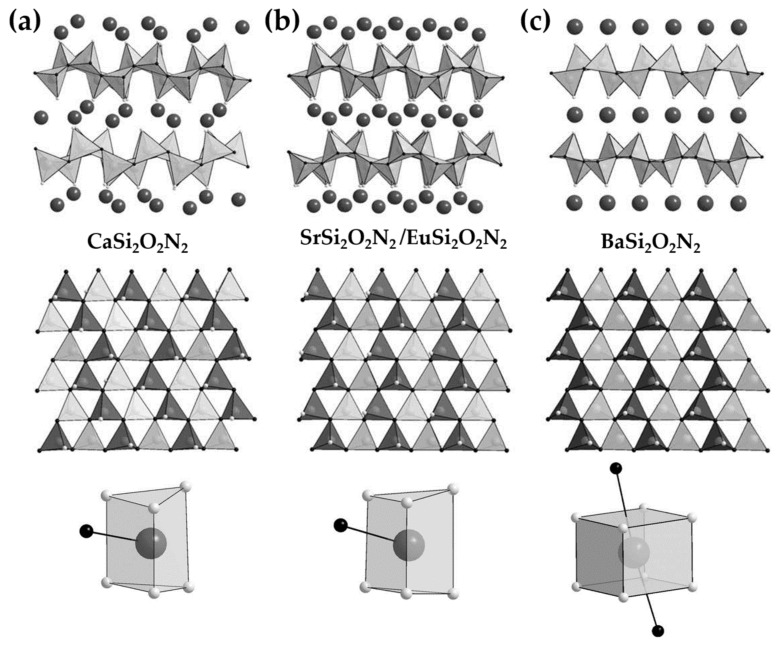
The crystal structure (top), silicate layers along [100] (middle) and cation coordination (bottom) in CaSi${}_{2}$O${}_{2}$N${}_{2}$ (**a**); SrSi${}_{2}$O${}_{2}$N${}_{2}$ (**b**); and BaSi${}_{2}$O${}_{2}$N${}_{2}$ (**c**). Tetrahedra with vertices up and down are depicted in dark gray, and in light respectively (N${}^{3-}$: black, O${}^{2-}$: light gray). Reproduced from Ref [241], Copyright (2009), with permission from Elsevier.

For MSi${}_{2}$O${}_{2}$N${}_{2}$:Eu${}^{2+}$ (M = Ba, Sr, Ca), the packing order of the polyhedra is more or less related to ML. In these three compounds, [SiON${}_{3}$] tetraheda are connected by sharing a vertex to form condensed layers, while cations are bound to the terminal oxygen of the tetrahedra in consecutive layers. They differ in the sequence of tetrahedra and coordination environment of cations. BaSi${}_{2}$O${}_{2}$N${}_{2}$ and SrSi${}_{2}$O${}_{2}$N${}_{2}$ (or EuSi${}_{2}$O${}_{2}$N${}_{2}$) have the same tetrahedron sequence, but the consecutive silicate layers in SrSi${}_{2}$O${}_{2}$N${}_{2}$ are shifted against each other to create distorted trigonal prisms of O atoms capped by one N atom (Figure 8b,c) [241]. On the contrary, CaSi${}_{2}$O${}_{2}$N${}_{2}$ adopts different tetrahedra sequences, even though Ca atoms occupy the same coordination polyhedra as those in SrSi${}_{2}$O${}_{2}$N${}_{2}$ (Figure 8a) [241]. The crystal structure of Sr${}_{1-x}$Ba${}_{x}$Si${}_{2}$O${}_{2}$N${}_{2}$: Eu${}^{2+}$ solutions, transform from triclinic (P1) to another triclinic (P1) and finally to orthorhombic as *x* increases, accompanied by a change of cationic coordination polyhedra. Accordingly, a drastic change of emission energy was observed [242,243], which was also found when pressure driven phase transition occurs in this solution [244]. The fact that both SrSi${}_{2}$O${}_{2}$N${}_{2}$ and BaSi${}_{2}$O${}_{2}$N${}_{2}$ shows ML, in contrast to CaSi${}_{2}$O${}_{2}$N${}_{2}$, suggests that the ordering of the tetrahedra plays a fundamental role in addition to the defects responsible for trapping electrons. In BaSi${}_{2}$O${}_{2}$N${}_{2}$:Eu${}^{2+}$, both ML and persistent luminescence spectra show a 4 nm shift compared to that of the steady state photoluminescence, which was argued to origin from the existence of crystal grains with both Pbcn and Cmc2${}_{1}$ [192]. The much lower intensity of ML in SrSi${}_{2}$O${}_{2}$N${}_{2}$:Eu${}^{2+}$ compared to BaSi${}_{2}$O${}_{2}$N${}_{2}$:Eu${}^{2+}$ is more likely to be related to differences in the concentration of traps [192] as thermoluminescence measurements revealed that SrSi${}_{2}$O${}_{2}$N${}_{2}$:Eu has a larger fraction of shallow traps than BaSi${}_{2}$O${}_{2}$N${}_{2}$:Eu${}^{2+}$ [190].

It is not possible to comment in detail the behavior of all ML phosphors in Table 1, but defects in phosphors are evidently present and playing an important role. Spinels, which show ML under low velocity impact, are found to have cationic disorders by co-existence of inverse spinels. A peculiar connectivity of polyhedra of anion framework renders CaZr(PO${}_{4}$)${}_{2}$ fairly anisotropic in thermal expansion [193]. The occurrence of multiple traps with different depth in CaZr(PO${}_{4}$)${}_{2}$: Eu${}^{2+}$ is likely to provide more channels to release trapped electrons under strains in the process of ML.

### 3.3. Microstructures

Optical properties of solid compounds are sensitive to both their general crystal structures and microstructures [245]. In addition to point defects and their agglomeration, there are still various kinds of microstructures, including twins, domains, domain walls, phase boundaries and compositional modulations. Moreover, the variation of chemical composition may generate unexpected ferroelectricity in intrinsically uniform compounds or by carefully doping distorted parts of the structure [246]. Polymorphisms and specific microstructures, if any, are listed in Table 2 for the purpose of searching potential elements that could impact the properties of ML compounds beyond their general crystal structure. The importance of atomic order/disorder, phase transitions and their consequences are also briefly outlined in this section.

#### 3.3.1. Structural Phase Transitions and Their Consequences

In general, a phase transition refers to the change from one homogeneous state of matter into another one under external influences such as temperature, pressure, electric or magnetic field, and chemical substitution, etc. Structural phase transitions in solids due to temperature or chemical substitution (solid solution) carry importance, being directly related to techniques of preparing phosphors or tuning their properties. They can be achieved by a slight displacement of atoms (displacive transitions), by the breaking and reconstruction of chemical bonds (reconstructive transitions), or by the rearrangement of atoms in an ordered fashion (order-disorder transitions) [273].

A displacive phase transition involves only slight alteration of bond lengths and their relative orientations, implying that the space groups of the two phases are both subgroups of the space group of a reference structure (or prototype). One of its fundamental features is the formation of non-homogeneously textured low-symmetry domain structures, which are separated by domain walls. The types of domains and domain walls can be deduced from group theory [274,275,276]. The role of domains or twins and their boundaries, as reported in many compounds in Table 2, cannot be underestimated in the sense that they attract the impurity atoms (dopants) or generate ferroelectricity which is not intrinsic to the matrix. For example, the ferroelastic domains in SrAl${}_{2}$O${}_{4}$ phosphor are pseudo-elastic under nano-indentation and are thought to help liberating trapped carriers in SrAl${}_{2}$O${}_{4}$:Eu${}^{2+}$, ultimately leading to ML [30]. Other important features of phase transitions are the anomalies of the elastic constants at the critical temperature T${}_{c}$ or pressure P${}_{c}$, where the crystal tend to strongly “soften” or “harden” [277]. ML near this temperature range will certainly reveal the dependency of ML on the elastic properties of the host.

**Figure 9:**
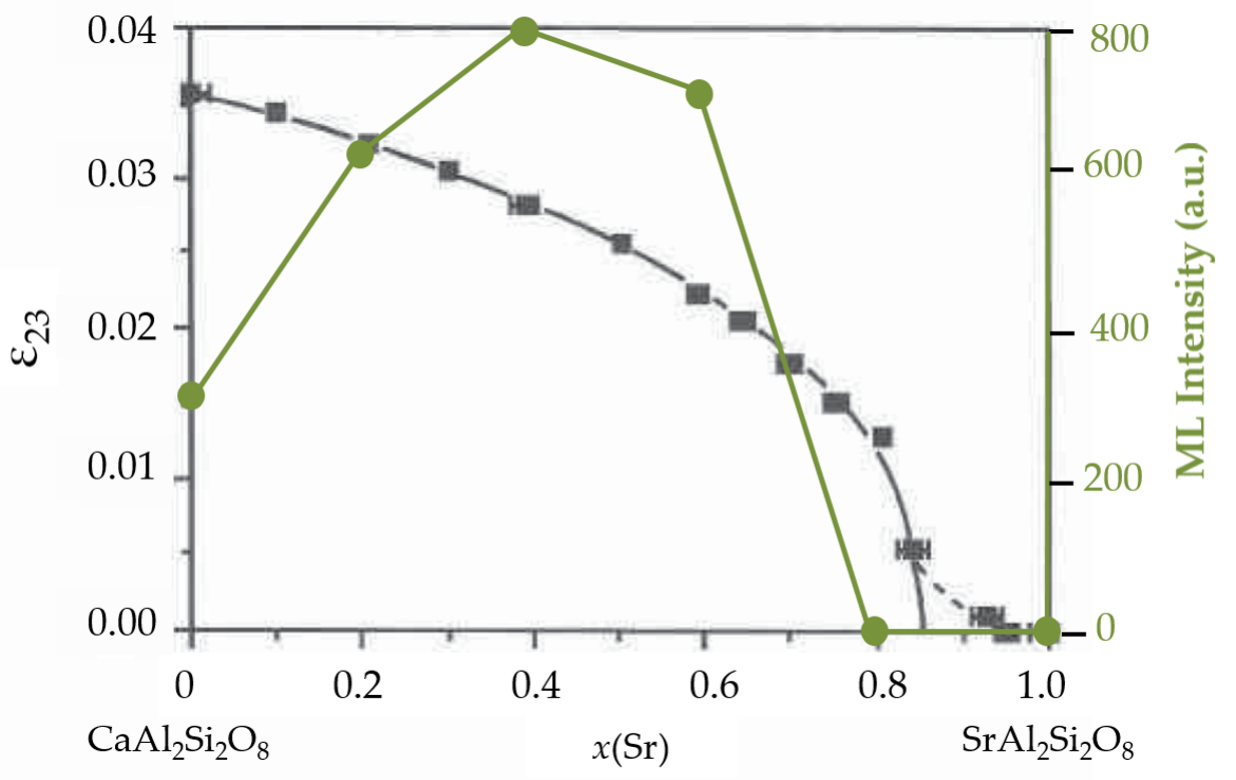
The order parameter—internal strain $\varepsilon_{23}$, and ML intensity data (deep green) as a function of Sr content in Ca${}_{1-x}$Sr${}_{x}$Al${}_{2}$Si${}_{2}$O${}_{8}$ series. Reproduced from Refs [170,216] with permission from Mineralogical Society of America and Copyright 2010, The Electrochemical Society, respectively.

Chemical substitutions can alter phase transitions of compounds. They expand or shrink the crystal lattice and thus tune the transition temperature, as demonstrated in (Ca${}_{1-x}$Sr${}_{x}$)${}_{2}$MgSi${}_{2}$O${}_{7}$ where substitution of strontium reduces the temperature of transition from commensurate to incommensurate phases [225,266]. Chemical substitution itself can also induce phase transitions, i.e., morphotropic phase transitions, and the boundary between composition ranges in a T-*x* phase diagram is often called morphotropic phase boundary (MPB). Morphotropic phase transitions give rise to effects that are related to internal strains and atomic ordering due to the different size and/or charge of the impurity atoms, such as the ordered occupation of Sr${}^{2+}$ in (Ca${}_{1-x}$Sr${}_{x}$)${}_{2}$MgSi${}_{2}$O${}_{7}$, Ca${}_{1-x}$Sr${}_{x}$Al${}_{2}$Si${}_{2}$O${}_{8}$, and Sr${}_{1-x}$Ba${}_{x}$Si${}_{2}$O${}_{2}$N${}_{2}$ as reviewed above. As predicted from theoretical calculations by the coupled cluster method, emission spectra of dopants in inorganic solids are predominantly influenced by the local coordination environment (up to the second nearest neighbours) [278]. Thus, cationic or anionic alloying can bring a drastic shift of emission energy if there is a drastic change of local structures. A gradual blue shift of emission spectra in Ca${}_{1-x}$Sr${}_{x}$Al${}_{2}$Si${}_{2}$O${}_{8}$:Eu${}^{2+}$ (0≤x≤1) [169] was due to a smooth variation of the local coordination environment with chemical substitution although its crystal phase from P$\bar{1}$ over I$\bar{1}$ to I$\frac{2}{c}$ [216]. On the contrary, the emission drastically shifts from blue to orange when *x* crosses 0.75 in Sr${}_{1-x}$Ba${}_{x}$Si${}_{2}$O${}_{2}$N${}_{2}$:Eu${}^{2+}$ solid solutions [242] because of a huge change in coordination polyhedra. The morphotropic phase transition in Ca${}_{1-x}$Sr${}_{x}$Al${}_{2}$Si${}_{2}$O${}_{8}$ (0≤x≤1) (Figure 9) causes the disappearance of internal strain at x= 0.86, around which the ML intensity drops to zero too in the Eu${}^{2+}$ doped phosphors. Another merit offered by MPBs is the possibility to enhance the piezoelectric coefficients near the MPB due to a maximized shear anisotropy of piezoelectricity [279,280]. An increased piezoelectric field is favourable to release trapped charge carriers to produce observable ML, assuming that piezoelectricity is the driving force for the release of trapped charge carriers.

#### 3.3.2. Modulated Structure and Chemical Gradients

Modulated structures are characterized by a periodic deformation of a basic structure that can be described by a conventional space group [34]. The modulation can be a periodic displacement of atomic positions or a periodic occupation of atoms at a specific crystallographic site [34], which can be interpreted in terms of the compromise between competing distortion modes or electronic instabilities [281]. A noteworthy case is the incommensurate structure in melilite compounds. A large displacement between the cations and the bridging O in tetrahedra layers results in distortions of the [MgO${}_{4}$] and [SiO${}_{4}$] tetrahedra. These distortions cannot be realized within one unit cell and thus lead to modulated structures [222,282].

**Figure 10:**
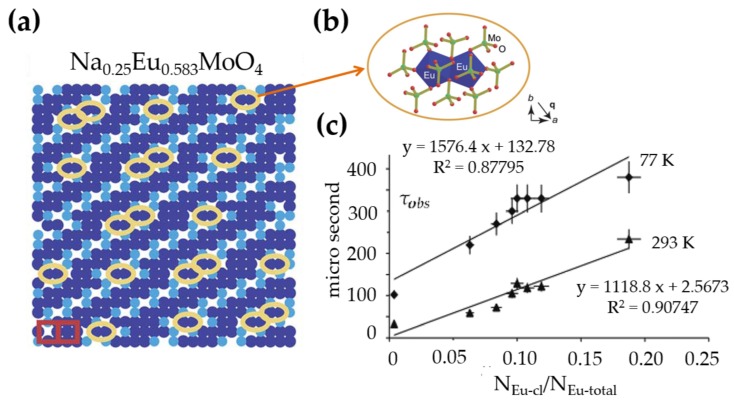
Part of the cation subset along [001] (**a**), the nearest neighbors of the Eu dimer (**b**) in Na${}_{x}$Eu${}^{3+}$O${}_{2/3-x/3}$MoO${}_{4}$, and (**c**) the lifetime of ${}^{5}D_{0}$ levels as a function of relative amount of Eu${}^{3+}$ clusters at 77 K and 293 K. (In (**a**), Eu${}^{3+}$: dark, Na${}^{+}$: light blue, vacancies: white, Eu${}^{3+}$-Eu${}^{3+}$: yellow, different cation distribution in adjacent cells: red box.) Reproduced from Ref [283] with permission of The Royal Society of Chemistry.

The structure or chemical gradients in modulated structures may generate polarization through flexoelectricity effects [43], as well as by attracting impurities onto domain walls or other structural gradients [246]. The structure fluctuations can impose interesting effects on optical properties which depend heavily on the local atomic environment. The scheelite compound Na${}_{x}$Eu${}^{3+}$O${}_{2/3-x/3}$MoO${}_{4}$ presents atomic occupational modulation when x< 0.5 (Figure 10a). Here, Eu${}^{3+}$ are attracted to form Eu${}^{3+}$-Eu${}^{3+}$ dimers (Figure 10b) due to the chemical gradient. The lifetime of the Eu${}^{3+}$ ${}^{5}$D${}_{0}$ emission, being closely connected to the luminescent quantum efficiency, depends on the fraction of Eu${}^{3+}$ ions that form dimers, as shown in Figure 10c [283]. Unfortunately, incommensurate structures in ML phosphors attract surprisingly little attention, although these structures can be important as local strain tends to modify the environment of dopants and hence the ML properties.

### 3.4. General Remarks

The relatively small number of ML compounds do not seemingly justify an explicit link between the crystal structures and the performance of ML, but it is true for most compounds that the emission centres of ML are the same as those of photoluminescence, persistent luminescence or electroluminescence, if present. The essential triggers for ML are found in the specific crystal structure, point defects and their agglomeration, and microstructures, such as domains or domain walls. Point defects and their clustering create suitable traps for charge carriers in phosphors. The change of their geometric configuration under strain brings about the change in binding energy for trapped charges, which may facilitate the escape of trapped charges. More importantly, centrosymmetry can locally be broken by defects and piezoelectricity can thus be observed [29]. In this regard, compounds with anisotropy in elasticity or piezoelectricity are favourable for the development of ML phosphors since internal electric fields in certain crystallographic directions can be large enough to induce observable ML. The structure survey shows roughly 80% of ML phosphors have flexible framework structures where anisotropy in the elasticity exists or can be expected.

Only limited research has been reported on ML at the microscale [58,149], but the influence of microstructure can be vital in several cases. Twin structures with various morphologies were found in SrAl${}_{2}$O${}_{4}$:Eu${}^{2+}$ phosphors and the domains show quasi-elastic character under nano-indentation (refer to Section 4.2 for their roles in ML mechanism). The mechanically assisted release of carriers from sites near boundaries was argued to induce ML in this compound [30]. Microstructures in ML phosphors clearly deserve more attention and deeper investigation.

## 4. Proposed Mechanism and Models

**Figure 11:**
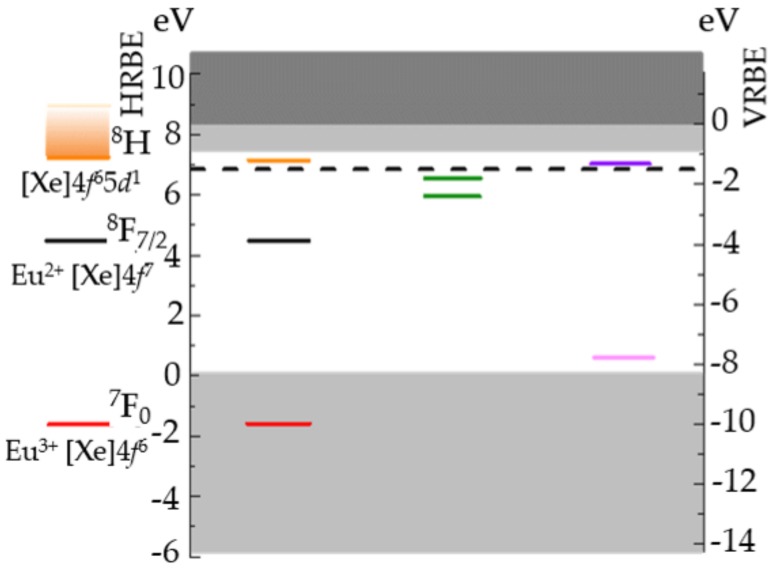
Vacuum referred (VRBE) and host referred (HRBE) binding energy of lanthanides R (R = Eu${}^{3+}$(red), Eu${}^{2+}$(black and orange), Dy${}^{3+}$(pink), Dy${}^{2+}$(purple)) and oxygen vacancies levels (green) with respect to the valence band of SrAl${}_{2}$O${}_{4}$ host, with all R being assumed to occupy the Sr1 site. Energy unit is eV. Reproduced with permission from Ref [92] Copyright (2006) American Chemical Society and from Ref [288] Copyright (2014) American Physical Society .

Evidently, processes in ML take place at several scales in time and space. It is necessary to overview possible mechanisms or dynamics of ML at different stages. At the scale of unit cells of ML phosphors, it is widely accepted that ML originates from transitions of charge carriers at traps. To illustrate the relationships between traps and the host, the ML phosphor SrAl${}_{2}$O${}_{4}$:Eu${}^{2+}$, Dy${}^{3+}$ serves an excellent example. The electronic structure of SrAl${}_{2}$O${}_{4}$ , as shown in Figure 11, is described by a band structure, with dopants (here Eu${}^{2+}$, Dy${}^{3+}$) treated as localized charge state transition levels. The exact eigenstates of the dopants (displaced along the side of the band diagram) are actually treated by crystal field theory. Intraconfigurational transitions, which are obtained by subtracting the total energy of final and initial states, can then be compared with experimental findings. Oxygen vacancies are likely to play a role in trapping electrons in SrAl${}_{2}$O${}_{4}$:Eu${}^{2+}$, Dy${}^{3+}$ since their levels are close enough to the conduction band [284]. The main influence of adding co-dopants, such as Dy${}^{3+}$, was argued to modify the trap depth distribution [83], stabilize vacancies via electrostatic interaction [92], or increase trap density via charge compensation mechanism [285] and trap carriers in some cases [286]. The positions of these defects levels are normally calculated by density functional theory. Interested readers are referred to Ref [287] for more information and the accuracy analysis.

The motion of large number of charge carriers (electrons or holes) often happens in phosphors at the spatial scale of microcrystals and the time span can be from 10${}^{-8}$ s to ∼100 h. Among many processes in charging stand out two kinds of activities that deserve special attention. On the one hand, 4f electrons of Eu${}^{2+}$ can be directly excited to its 5d states and subsequently ionizes (i.e., Eu${}^{3+}$ is obtained), which was confirmed by a Eu${}^{2+}$→ Eu${}^{3+}$ process in XAFS [286] and EPR [289] results. The ionization can be thermally assisted, especially when one excites the lower 5d states of Eu${}^{2+}$. On the contrary, the Dy${}^{3+}$→ Dy${}^{2+}$ was not convincingly detected, which suggested that the electron is presumably not fully trapped by Dy${}^{3+}$ although loosely bound Dy${}^{3+}$-e pairs are also possible [286]. On the other hand, electron-holes can be created by elevating electrons from the valence band to the conduction band, which can be trapped at various defects (donors for electrons, acceptors for holes), but only electrons were argued to be the carriers responsible for the subsequent luminescence [289]. For other ML phosphors, holes can be charge carriers.

The dynamics of these processes can be approximately simulated in the framework of homogeneous or heterogeneous chemical reaction methods, where the change of species are described by differential equations. When the dependency on spatial distances are included, one can reach numerical solutions based on the Monte Carlo method, see Refs [290,291] for example. Another route to ML dynamics lies in the non-equilibrium thermodynamics, in which the evolution of all admissible processes can be predicted by the second law of thermodynamics. The irreversible processes (ML here), can be related to stress/strain tensor, or its related variables. The intrinsic irreversibility of dynamic deformation here can be treated as “damage” to the electrons stored at defects and thus can be included in thermodynamics as an internal variable. It is possible to decouple processes at different length/time scale via these phenomenological approaches and its validation with experiments.

However, we are only able to detect ML at the scale that statistical mechanics works well, which usually provides the average value of intrinsic ML properties. The driving force of ML should be a small part of the distortion energy during dynamic processes since spatial dependency of ML agrees well with the Von Mises stress $\sigma_{v}=\sqrt{\left[{\left(\sigma_{1}-\sigma_{2}\right)}^{2}-{\left(\sigma_{2}-\sigma_{3}\right)}^{2}-{\left(\sigma_{1}-\sigma_{3}\right)}^{2}\right]/2}$, which is associated with the asymmetry of principal stress $\sigma_{i}\;\left(i=1,2,3\right)$ [292]. Unfortunately, Von Mises stress is not a tensor that can be used as a state function of the ML phosphor system. In reality, we are not relating stress (a state function) to ML, but its change, which might include non-linear effects of ML intensity. Clearly, we missed the link that relates mechanics to the real driving force of ML, which often takes place at the scale of single grain particles of ML phosphors.

The best scenario is to have a good knowledge of the relevant defects level and how they change under mechanical load, so that we then can connect experimental findings to defects in phosphors through thermodynamics. The intuitive concept is that the energy barriers of trapped carriers are lowered upon mechanical load, either by reducing the band gap of the host or changing the positions of defects in the band gap of the host. For many wide band gap semiconductors, the band gap changes about 10–40 meV under hydrostatic pressure of 1 GPa or at 1% strain of lattice [293] (*The band gap of semiconductors under pressure p can be written as* $E_{g}\left(p\right)=E_{g}\left(0\right)+a\cdot p+b\cdot p^{2}$*,* $E_{g}$ *in eV and p in GPa. The coefficient a can reach ∼50 meV/GPa for many direct wide band gap semiconductors and about −20 meV/GPa for indirect band gap semiconductors. b is on the order of 0.1 meV/GPa*${}^{2}$). In a typical ML experiment, the band gap reduction is estimated as several meV since the maximum shear stress is often below 100 MPa (Appendix A). External electric field can only cause a tiny reduction of band gap via the Franz-Keldish effect [294,295] or quantum confined Stark effect [296] of quantum well structures (e.g., 0.7 meV for hexagonal ZnS crystal [297] at 10${}^{4}$ V/cm). Another source of band gap shift is from extended defects in crystal such as dislocations [298], faults and grain boundaries, which can generate large electric fields (10${}^{5}$ V/cm for ZnS [299]) and thus also reduce the band gap. In this regard, piezoelectric phosphors are likely to have appreciable reduction of band gap when they are strained, which coincides with the fact that many ML phosphors exhibit defects as stated in Table 2. It is also very likely that energy levels of defects move towards to conduction band because of the geometry change of the configuration, which is probably due to its relatively distorted structure of the phosphors.

### 4.1. Mechanism 1: Piezoelectrically Induced Detrapping by Reducing Trap Depth

This mechanism is based on the idea that piezoelectricity is responsible for the internal electric field that helps to release trapped carriers, which takes place in persistent phosphors or doped semiconductors. Piezoelectricity can originate from intrinsic properties of non-centrosymmetric compounds (excluding compounds with 432 point group) or centrosymmetric compounds that have proper types of lattice defects or microstructure [27,28,262]. A decrease of effective energy barriers of traps leads to the release of an extra amount of carriers with the help of mechanical load [189]. Take Eu${}^{2+}$ as a typical emitting center, and this mechanism is summarized in the following steps:

i upon photo-excitation, 4f electrons of Eu${}^{2+}$ ions are lifted to 5d levels and subsequently escape to the conduction band, leading to Eu${}^{2+}$ ions being oxidized to Eu${}^{3+}$;
ii the created electrons in the conduction band are trapped at defect centres, e.g., vacancies $V_{O}^{‥}$, co-dopants R${}^{3+}$, or other defects;
iii when stress is loaded, the depth of traps is reduced due to the piezoelectric field, leading to the detrapping of electrons (to the conduction band);
iv the released electron is captured by Eu${}^{3+}$, which in turn reduces to an excited ion Eu${}^{2{+}^{*}}$;
v de-excitation of the excited Eu${}^{2{+}^{*}}$ ions provides emission of light.

Interested readers are referred to references [28,300,301,302,303] for a summary. It should be straightforward that anisotropy in the elasticity is favourable here to lower site symmetry or to create strong electric field in specific directions. Moreover, the energy levels of defects and that of dopants should be close enough if a direct tunneling between them is responsible for ML, e.g., in Ca${}_{2}$Nb${}_{2}$O${}_{7}$:Pr${}^{3+}$ [29]. Actually, this mechanism seems to work well for most, if not all, persistent phosphors where trap depths are in a proper range.

However, it is more complicated to apply this mechanism to ML in some doped semiconductors, especially ZnS:Mn${}^{2+}$ and CaZnOS:Mn${}^{2+}$, in which the traps are probably emptied at room temperature. ML in ZnS:Mn${}^{2+}$ was argued to originate from electroluminescence triggered by internal piezoelectric field since traps are too shallow [106] to hold electrons at room temperature. The maximum piezoelectric field of perfect ZnS crystal is estimated as only 320 V/cm in a typical ML experiment (Appendix C), which is about 100 times smaller than the threshold field for electroluminescence [304]. In fact, the threshold of stress for ML in ZnS:Mn${}^{2+}$ is only 0.6 MPa [108] , which corresponds to an electric field of 2 V/cm. Chandra et al. proposed that enhanced piezoelectric constants can be achieved by electric dipoles induced in photo-excitation [305], but the existence and stability of such dipoles still remains speculative. For the case of CaZnOS:Mn${}^{2+}$ phosphor, the red emission is attributed to ${}^{4}$G(${}^{4}$T${}_{1}$)→${}^{6}$S(${}^{6}$A${}_{1}$) transition in 3d${}^{5}$ states of Mn${}^{2+}$, which can be excited through charge transfer states efficiently [306]. Its ML intensity is independent of prior heat treatment under different temperature, suggesting that traps for ML are either too deep or a novel mechanism should be proposed. Huang et al. proposed that intrinsic defects produced by substituting Zn/Ca with Mn in the lattice are responsible for ML and concentration quenching [307]. Interestingly, oxygen vacancies are claimed to be responsible for the luminescence process in undoped CaZnOS [133]. Consequently, the complexity of defects and the interaction with piezoelectricity of hosts can tremendously complicate our understanding of ML.

It is interesting to note that, when multiple defects and luminescent centres are both active for ML, a tuning of emission colour could be anticipated via strain/stress. This is true for ZnS:Al${}^{3+}$, Cu${}^{+}$ whose emission spectrum shifted to higher energy when the frequency of the stress field [121], the electric field [121,123] or the magnetic field [125] increases. It seems that some deeper traps are only available for luminescence under dynamic load with high strain rate.

To obtain a quantitative model, one must have a good knowledge of the distribution of traps with trap depth, how the reduction of trap depth relates to stress and the dynamics of free carriers in a host’s conduction band. Luckily, several empirical models are available to provide a predictive relation between mechanical load and instantaneous ML intensity.

#### 4.1.1. The Rate Equation Method

The dynamics of luminescence can be often modelled by solving “rate equations”, where the emission of a photon is considered as a homogeneous “chemical reaction”. Kim et al. managed to numerically solve rate equations for SrAl${}_{2}$O${}_{4}$:Eu${}^{2+}$, Dy${}^{3+}$ in an attempt to understand how stress helps to release trapped carriers [308]. Unfortunately, the original rate equations were falsely based on an assumption that holes are the charge carriers and that Dy${}^{4+}$ is formed [77]. In fact, electrons are the charge carriers in this particular phosphor and oxygen vacancies are presumably electron traps. Hence we display here the correctly labelled rate equations using the symbols V and V${}^{*}$ to denote traps before and after capturing electrons. The modified rate equations are,

(9) $$\frac{dN_{Eu^{2{+}^{*}}}}{dt}=K_{T}\frac{d\sigma }{dt}N_{Eu^{3+}}N_{V^{*}}-K_{P}N_{Eu^{2{+}^{*}}}$$

(10) $$\frac{dN_{Eu^{2+}}}{dt}=K_{P}N_{Eu^{2{+}^{*}}}$$

(11) $$\frac{dN_{Eu^{3+}}}{dt}=\frac{dN_{V^{*}}}{dt}=-\frac{dN_{V}}{dt}=-K_{T}\frac{d\sigma }{dt}N_{Eu^{3+}}N_{V^{*}}$$

where K${}_{T}$ and K${}_{P}$ are rate constants of detrapping and recombination respectively, and *N* is the numbers of ions or vacancies per unit volume. The initial conditions and boundary condition are, respectively,

$$N_{Eu^{2{+}^{*}}}=0,N_{Eu^{2+}}=N_{V}=N_{0}\left(1-m\right)/2,N_{Eu^{3+}}=N_{V^{*}}=N_{0}m/2,\;\mathrm{at}\;t=0;$$

$$N_{Eu^{2+}}+N_{Eu^{2{+}^{*}}}+N_{Eu^{3+}}+N_{V^{*}}+N_{V}=N_{0},\;\mathrm{at}\;\mathrm{any}\;\mathrm{time}\;t.$$

Here, $N_{0}$ is the total number of ions or vacancies both at ground state and excited state and *m* is the initial ratio of traps that has already captured electrons. The quantity $N_{Eu^{2{+}^{*}}}$, which is supposed to be proportional to the spontaneous ML intensity, can be solved by a Runge–Kutta algorithm [309].

**Figure 12:**
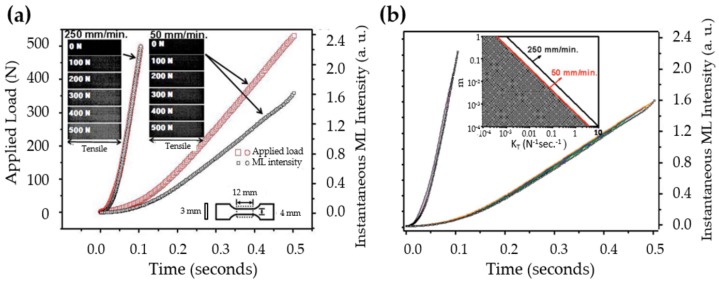
(**a**) The ML intensity and applied load for two different loading rates; and (**b**) their comparison with calculated results (colour line) where the inset shows boundaries of *m* and $K_{T}$ for downward convexity of ML. Reproduced from Ref [308], with permission. © 2009 Optical Society of America.

For a rod (dimension shown in the insect of Figure 12a) subjected to a maximum of 500 N applied at two different rates, the instantaneous ML intensities were obtained (Figure 12a) and compared with numerical solution of rate equations (Figure 12b). The main feature of ML curve (downward convexity) is only present when the combination of *m* and $K_{T}$ falls into a specific region as shown in the inset of Figure 12b. Clearly, *m* is related to the photoexcitation process while $K_{T}$ depends on the properties of traps and the release mechanism of electrons.

Similarly, Chandra et al. proposed that instantaneous ML intensity is proportional to the number of electrons in conduction band $n_{e}^{*}$ [310,311], which follows from the rate equation,

(12) $$\frac{dn_{e}^{*}}{dt}=-\frac{\partial n_{e}\left(E_{trap}\left(\sigma \right)\right)}{\partial t}-\sum_{i}n_{e}^{*}/\tau_{i}$$

in which $n_{e}$ is number of trapped electrons at defects, $E_{trap}$ the depth of a trap and $\tau_{i}$ the lifetime of excited electrons in conduction band after which they are retrapped or captured by Eu${}^{2{+}^{*}}$ centres via process *i*. They further assumed that trap density decays exponentially with trap depth and that the increase of electrostatic energy of trapped electrons is responsible for the reduction of detrapping barriers, i.e., $\frac{\partial E_{trap}}{\partial t}=\frac{\partial W}{\partial t}$ with $W=\psi {\left(d_{0}e/\varepsilon_{r}\right)}^{2}\sigma^{2}$ the electrostatic energy of a trapped electron due to stress σ. Here, $d_{0}$ is the piezoelectric coefficient, $\varepsilon_{r}$ the dielectric constant, *e* the elementary charge, and ψ a scale factor. Then, the detrapping rate of electrons due to stress is given by:

(13) $$-\frac{\partial n_{e}\left(E_{trap}\left(\sigma \right)\right)}{\partial t}=\psi n_{0}Z^{2}{\left(d_{0}e/\varepsilon_{r}\right)}^{2}\sigma \frac{\partial \sigma }{\partial t}\exp\left[Z{\left(d_{0}e/\varepsilon_{r}\right)}^{2}\sigma^{2}\right]$$

A final solution provides formulae for the instantaneous intensity $I_{ML}\left(t\right)$ and accumulated ML intensity $I_{ML}\left(T_{t}\right)$ as:

(14) $$I_{ML}\left(t\right)=\frac{2n_{0}Z^{2}\psi {\left(d_{0}e/\varepsilon_{r}\right)}^{2}}{\sum_{i}1/\tau_{i}}\sigma \frac{\partial \sigma }{\partial t}\exp\left[-Z\psi {\left(d_{0}e\varepsilon_{r}\right)}^{2}\sigma^{2}\right]$$

(15) $$I_{ML}\left(T_{t}\right)=\int_{0}^{T_{t}}I_{ML}\left(t\right)dt=\frac{Zn_{0}}{\sum_{i}1/\tau_{i}}\left\{1-\exp\left[-Z\psi {\left(d_{0}e/\varepsilon_{r}\right)}^{2}\sigma^{2}\right]\right\}$$

Obviously, the instantaneous ML intensity is proportional to the product of stress and stress rate while accumulated ML intensity is roughly quadratic with stress, which also agrees with experimental findings [81]. Actually, the assumption of the trap distribution is not physically validated, e.g., a Gaussian distribution [312] was found in CaAl${}_{2}$O${}_{4}$:Eu, Nd, and should not be a prerequisite in deducing the above equation. Furthermore, the bimolecular nature of retrapping and recombination, which is valid for persistent luminescence and optically stimulated luminescence [313], was absent in Chandra’s formulation and can lead to failure of describing the hysteresis behaviour of ML (Section 4.1.2).

#### 4.1.2. Viscoelasticity Method

**Figure 13:**
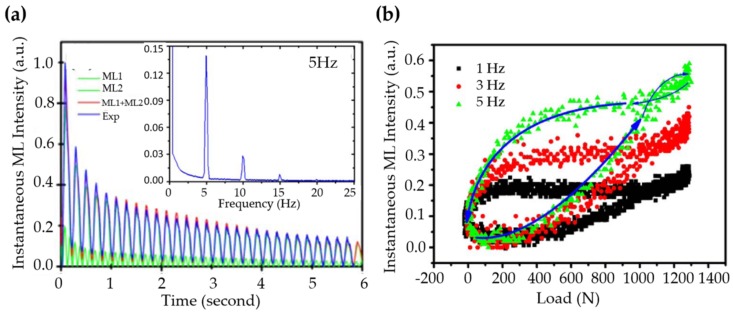
(**a**) Fitting of the ML intensity with a model with loading frequency of 5 Hz (the inset shows the corresponding FFT results), and the hysteresis loops (**b**) at various frequencies (1–5 Hz) of SrAl${}_{2}$O${}_{4}$:Eu${}^{2+}$/Dy${}^{3+}$ composites. Reproduced from Ref [314], with permission. © 2014 Optical Society of America.

It is possible that instantaneous ML intensity also depends on the history of loading, which often exhibits a phase lag with respect to the load and a hysteresis effect. Under cyclic sinusoidal loads, the intensity of instantaneous ML and its associated persistent luminescence of SrAl${}_{2}$O${}_{4}$:Eu${}^{2+}$, Dy${}^{3+}$ shows a phase lag with respect to the load (Figure 13a) and a hysteresis effect during load-unload cycles (Figure 13b), which is intrinsic to ML processes since no phase lag was detected in the stress-strain relationship [314]. Therefore, Sohn et al. included a “second-order” term to account for the non-linearity of ML [314] so that:

(16) $$I_{ML}\left(t\right)=\left[\alpha \sigma \left(t\right)+\beta \sigma {\left(t\right)}^{2}\right]D\left(t\right)$$

in which $D\left(t\right)$ describes the decay of light intensity and α, β are complex constants accounting for the phase lag or hysteresis. An excellent fitting of experimental data can be obtained through this model, as shown in Figure 13a.

A more detailed treatment on the hysteresis of ML phenomenon was given by Dubernet et al., who built the model from linear viscoelasticity theory [315]. The mechanical power taken by the SrAl${}_{2}$O${}_{4}$:Eu${}^{2+}$, Dy${}^{3+}$ phosphor depends on the history of applied strain energy,

(17) $$f\left(t\right)=|\frac{d}{dt}\int_{0}^{t}\varphi \left(t-s\right)\frac{\partial \left[\sigma^{2}\left(s\right)/E\right]}{2\partial s}ds|$$

where φ is a creep function $\left(\dot{\varphi }\geq 0,\varphi \left(t\leq 0\right)=0,\varphi \left(t\to \infty \right)=1\right)$. $\varphi \left(t\right)$ can be calculated from a relaxation function, which is a stretched exponential function for complex system with strong correlation [316,317], and ends with,

(18) $$\varphi \left(t\right)=1-\exp\left[-{\left(t/\tau \right)}^{\beta }\right]$$

The detrapping rate of trapped electrons ($\frac{N_{dc}}{dt}$) was assumed to obey the same stress dependence as $f\left(t\right)$ and to be proportional to the relative fraction of the remaining trapped electrons, which leads to

(19) $$\frac{dN_{dc}}{dt}=\alpha f\left(t\right)\frac{N_{tc}-N_{dc}}{N_{tc}}$$

where $N_{dc}$ and $N_{tc}$ are the number of total and of trapped electrons, respectively. The instantaneous ML intensity at *t* should be proportional to the rate of photon emission ($\frac{dN_{dc}}{dt}$), thus

(20) $$I_{ML}\left(t\right)=\psi f\left(t\right)\exp\left[-\frac{\alpha }{N_{tc}}\int_{0}^{t}f\left(s\right)ds\right]$$

with ψ a scale factor.

**Figure 14:**
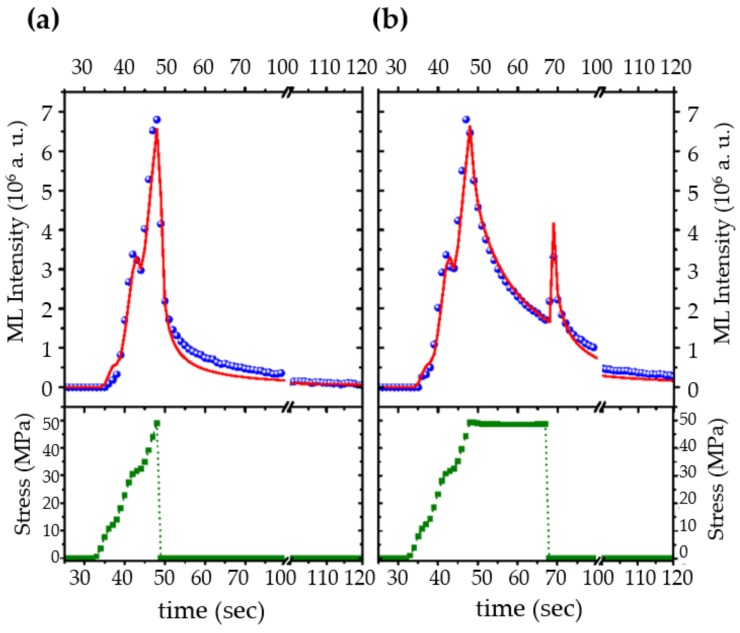
ML intensity (top, blue circles) for varying loads (bottom, green) without (**a**) and with (**b**) holding at maximum loading for some time in the glass composite containing SrAl${}_{2}$O${}_{4}$: Eu${}^{2+}$, Dy${}^{3+}$, and their fitting by the model (top, red lines). Reproduced from Ref [315], with the permission of AIP Publishing.

As shown in Figure 14, an excellent agreement was achieved between ML intensity and the fitting of the model under varying loading rate. The ML peak upon abrupt decrease of load was also predicted by this model, while its physical picture can be understood from the Boltzmann superposition principle [53]. The abrupt release in load is equal to the sum of stress before unloading plus an equal amount of load in the negative direction, which induces recovery phenomena (see Figure 2). In our case, it produces another ML peak. The physical origin of hysteresis of ML are argued to be the combination of trapping and retrapping of electrons during ML since the phase lag and hysteresis loop disappear upon illuminating the material with a laser of high power [318].

### 4.2. Mechanism 2: Carrier Release by the Electric Field Produced by Domain Structures

**Figure 15:**
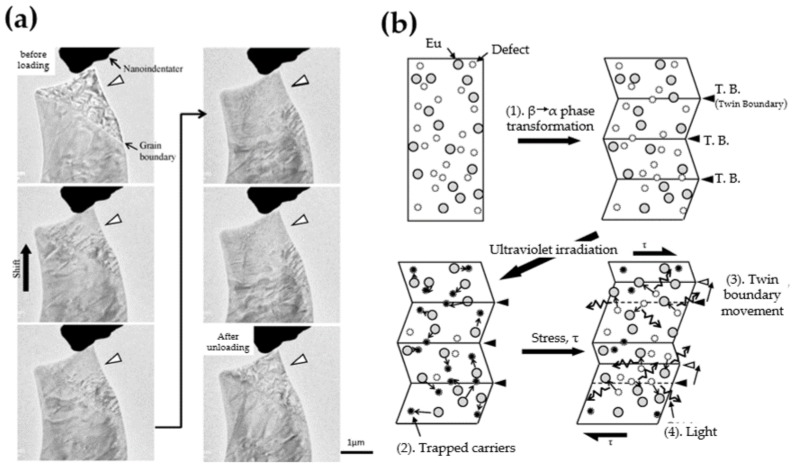
Snapshots of a domain boundary (**a**) in SrAl${}_{2}$O${}_{4}$: Eu${}^{2+}$ during nano-indentation, and the schematic diagram (**b**) showing the proposed mechanism. Reproduced from Ref [30], Copyright (2013), with permission from Elsevier.

As reviewed in Section 3.3, domain structures possess special mechanical properties and they can also create internal electric fields. Indeed, domains and domain walls were proposed to be linked to ML by several researchers [30,175]. Matsuo et al. reported the existence of twin structures with three different morphologies formed by the thermoelastic martensitic transition in SrAl${}_{2}$O${}_{4}$:Eu${}^{2+}$ [30]. The movement of twin boundaries under nano-indentation exhibits pseudo-elastic behaviour (shown in Figure 15a). The authors then proposed a model based on this unique behavior of twin boundaries as follows (Figure 15b):

i electrons are photo-ionized from Eu${}^{2+}$ ions and trapped at defects in SrAl${}_{2}$O${}_{4}$:Eu${}^{2+}$.
ii upon the mechanical load, the twin boundaries show a pseudo-elastic deformation and creates an electric field around the boundary to release the trapped electrons.
iii the electrons are captured by Eu${}^{3+}$, which turn into Eu${}^{2{+}^{*}}$, and the de-excitation to the ground state of Eu${}^{2+}$ yields the emission of light.

When boundaries meet with Eu${}^{3+}$ ions, electrons at boundaries can reduce Eu${}^{3+}$ into Eu${}^{2{+}^{*}}$. Since there are many kinds of microstructures in SrAl${}_{2}$O${}_{4}$:Eu${}^{2+}$, other defects could also play a role. Unfortunately, the scarcity of quantitative data has prevented researchers from building a mathematical formula to validate the mechanism proposed here.

Wang et al. proposed that the ferroelectric domains in composite ceramics of Pr${}^{3+}$-doped BaTiO${}_{3}$-CaTiO${}_{3}$ induce large electrostriction effects [237], electroluminescence and ML [175]. The composite material Ba${}_{1-x}$Ca${}_{x}$TiO${}_{3}$ (0.25<x<0.9) was thought to be composed of the tetragonal ferroelectric phase Ba${}_{0.77}$Ca${}_{0.23}$TiO${}_{3}$ and orthorhombic normal dielectric Ba${}_{0.1}$Ca${}_{0.9}$TiO${}_{3}$, which has a relative dielectric constant of 160–200 [175]. The polarization of Ca-rich phases interacts with the ferroelectric domains in Ba-rich phases since the two phases contact in three dimensions. The rotation of domains is thus hampered, resulting in a high electrostriction effect near the solution limit. Under mechanical load, ferroelectric phases provide electric fields, which become enhanced by electrostriction, and can then excite luminescent centres Pr${}^{3+}$ in the persistent luminescent phosphor Ba${}_{0.1}$Ca${}_{0.9}$TiO${}_{3}$:Pr${}^{3+}$, leading to luminescence.

The study of ML at a microscale is not readily available and the direct relationships between domains and light emission have thus not yet been substantiated. The mechanism proposed here provides clues that the hysteresis of ML might be related to domain states and other microstructures.

### 4.3. Mechanism 3: Carrier Release by Movement of Dislocation

Defects in coloured alkali halides can be grouped into F-centres (an electron trapped at a anion vacancy), V${}_{K}$-centres (*A V*${}_{K}$*-centre is the self-trapped hole in deformed alkali halides, i.e. a combination of a neutral halogen atom (the hole) with an adjacent halide anion, both off their regular site. It moves only by incoherent jumps from one site to another due to the local lattice distortion and polarization. See Ref [319]*), and impurity centres (such as Cu${}^{+}$, Tl${}^{+}$, Ag${}^{+}$ etc.) [320,321]. The energy levels of F-centres and V${}_{K}$-centres are often several hundreds of meV below the conduction band and above the valence band, respectively [320,321]. Shallow electron traps can be present as well. Charged by cations or anions, dislocations can bind electrons, holes and excitons [322] through elastic [323] and electrostatic [324] interactions with point defects . The interactions are more predominant when the dislocations are bent or move during elastic and plastic deformation, respectively.

It has now been established that ML of coloured cubic alkali halides is mainly due to the recombination of electrons from F-centres and holes from V${}_{K}$-centers or impurities [64,67,325], as exceptionally pure and additively coloured crystals do not yield light due to the absence of holes [66]. The bending of dislocations due to internal friction [326] within the elastic limit interacts with electrons in colour centres, reducing the energy barriers for electrons. Upon plastic deformation, a dislocation line moves as a whole in crystals and helps to liberate electrons when it sweeps across F-centres with the assistance of thermal energy. The liberation of holes from V${}_{K}$-centres or impurities can also be sensitized by dislocations [327]. The existence of dislocation induced bands (DIBs) in the band gap is not always valid, for example such DIBs are absent in the band gap of KCl and NaCl as predicted by quantum chemical calculations [328]. The energy difference between the F-centre levels and DIBs as claimed in temperature dependent ML is actually associated with the thermal activation energy of the movement of dislocations [329]. The driving force of capturing carriers (electrons or holes) is more probably related to the geometrical configuration, density and velocity of dislocations, which also determines the activation energy of dislocation movement. Vividly enough, the anisotropic distribution of ML on certain crystal facets of rock salt compounds coincides with the geometrical alignment of thier slip systems [64], indicating slip of dislocation is rather essential in ML.

**Figure 16:**
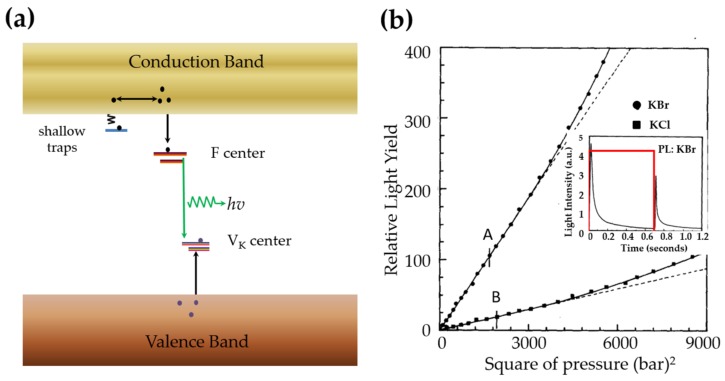
Schematic diagram (**a**) depicted the ML induced by movement of dislocation, and the ML intensity (**b**) as a function of square of pressure of KBr and KCl (A, B indicate their elastic limit and red rectangle in the insect indicates the load history). Reproduced from Ref [330], Copyright (2010), with permission from Elsevier and Ref [66], Copyright (1982), with permission from Elsevier, respectively.

A schematic diagram is presented in Figure 16a as modified from Ref [330]. It should be pointed out that carriers responsible for ML originates from F-centres and V${}_{K}$-centres and also from the movement of dislocations. The black arrows pointing to F-centre and V${}_{K}$-centre represent the capturing of produced carriers while carriers recombine mostly via the conduction or valence bands of host. Chandra et al. developed a mathematical model for the evolution of the ML intensity during elastic and plastic deformation [326,330] under a step load, which was approximated by,

(21) $$\sigma \left(t\right)=\sigma_{0}\left[1-\exp\left(-t/\tau_{st}\right)\right]$$

with $\sigma_{0}$ being the maximum amplitude of stress and $\tau_{st}$ the rise time of the stress. The authors assumed that the bending of a dislocation will sweep some area within the crystal as the movement of dislocations does. Therefore the difference between elastic and plastic deformation is reflected in the choice of the stress-strain relation, i.e., Hooke’s law for elastic deformation and the power-law for plastic deformation [330]. The rate of dislocations swept out to the surface is

(22) $$\frac{dS}{dt}=N_{m}\upsilon_{d}$$

where $N_{m}$, $\upsilon_{d}$ are the number and velocity of moving dislocations respectively. During its movement, dislocations capture electrons with a rate,

(23) $$\frac{dn_{d}}{dt}=\alpha^{\prime }n_{F}r_{F}\frac{dS}{dt}-\frac{n_{d}}{\tau_{d}}$$

in which $n_{d}$ is the electron density generated in the dislocations and $\alpha^{\prime }$ a constant. $n_{F}$ and $r_{F}$ are the density and the interaction radius of the F-centres. The recombination of these electrons with holes at V${}_{K}$-centres induces ML, whose instantaneous intensity should be proportional to $n_{d}$. Given the stress-strain relationship, $N_{m}$ can be calculated by:

(24) $$\frac{dN_{m}}{dt}=\beta \frac{d\varepsilon }{dt}-\frac{N_{m}}{\tau_{\sigma }}$$

in which β and $\tau_{\sigma }$ are constants. The solution of $n_{d}$ yields the following form for both elastic and plastic deformation:

(25) $$n_{d}\left(t\right)=c_{1}\left[\exp\left(-t/\tau_{1}\right)-\exp\left(-t/\tau_{2}\right)\right]+c_{2}$$

where $c_{1}$, $c_{2}$, $\tau_{1}$ and $\tau_{2}$ are constants. The solution shows first an increase and then a decrease of the ML intensity as a function of time, which agrees fairly well with experimental findings. In the case of deformation under fixed strain rate, Chandra *et al.* took into account the diffusion and drift of electrons in dislocations, and obtained a single exponential relaxation function [331]. However, the ML peak upon the abrupt release of the load (Figure 16b) was not predicted by the models above and it may be related to the viscoelastic nature of ML in alkali halides. Other experimental evidence that cannot be explained by this model is the fact that the instantaneous ML intensity is proportional to the product of strain and strain rate [66], and that it is inversely proportional to temperature [332] with an activation energy on the order of ∼0.1 eV regardless of the chemical composition [72] in various alkali halides.

Hayashiuchi et al. proposed another model to explain experimental findings by considering not only the contribution of electrons and holes liberated by the movement of dislocation, but thermal energy as well [333]. To calculate the contribution of electrons to instantaneous ML intensity, rate equations for the number of free electrons (*n*) and electrons at the F-centre (*m*) were respectively:

(26) $$\frac{dn}{dt}=\sum_{x,m}\left(x_{m}\chi_{m,m-1}-x_{m}\chi_{m,m+1}\right)$$

(27) $$\frac{dx_{m}}{dt}=x_{m-1}\chi_{m-1,m}+x_{m+1}\chi_{m+1,m}-x_{m}\chi_{m,m+1}-x_{m}\chi_{m,m-1}$$

in which $x_{i}$ is the density of F-centres with charge *i*, and $\chi_{i,j}$ denotes the transition probability from state *i* to state *j*. The number of photons emitted from $x_{m}$ per unit time $I_{xm}$ is thus:

(28) $$I_{xm}=Cx_{xm}k_{xm}n$$

with $k_{xm}$ the product of the electron capture cross section, electron velocity, and total number of F-centres before deformation (C). As n≪1 , the ML from V${}_{K}$-centres was given by,

(29) $$I_{ML}≃C_{V}{\left(\frac{k_{V}k_{V}}{k_{F}k_{F^{+}}}\right)}^{1/2}{\left(\chi_{F^{-},F}\chi_{F,F^{+}}\right)}^{1/2}$$

provided that the cross section for electron capture is small and the density of V${}_{K}$-centres is large. After introducing the rate of point defects interacting with dislocations, $p≃\pi r^{2}\rho$ with ρ the dislocation density and *r* the effective interaction range, an expression could be obtained:

(30) $$I_{ML}≃C_{V}{\left(\frac{k_{V}k_{V}}{k_{F}k_{F^{+}}}\right)}^{1/2}\frac{p}{\tau }$$

in which τ is a relaxation time (close to the pinning time of dislocations), being largely determined by thermal activation. Let *U* be the potential barrier of dislocation movement and Ω the activation volume, then the depinning probability per unit time $\frac{1}{\tau }$ is given by,

(31) $$\frac{1}{\tau }=\frac{1}{\tau_{0}}{\left(\frac{\dot{\varepsilon }}{\dot{\varepsilon_{0}}}\right)}^{a}\exp\left(-\frac{U^{\prime }}{kT}\right)$$

Here, $U^{\prime }$ is defined as

$$U^{\prime }=\left(1-\frac{\langle U\rangle \Omega }{U\langle \Omega \rangle }\right)U,\;\mathrm{and}\;a=\frac{\langle \Omega \rangle }{\Omega }$$

in which 〈〉 means average. A final relationship between strain rate and ML intensity was thus established :

(32) $$I_{ML}\propto {\dot{\varepsilon }}^{a}\exp\left(-\frac{U^{\prime }}{kT}\right)$$

This explained the power-law dependence of the instantaneous ML intensity on strain rate and the Arrhenius relation between instantaneous ML intensity and temperature. When $\langle U\rangle ∼U$ and $\langle \Omega \rangle ∼\Omega$, where a≃1, the temperature dependence of $I_{ML}$ is weak and $I_{ML}\propto \dot{\varepsilon }$.

### 4.4. Remarks

For most of the investigated materials, mechanisms of ML are far from complete. New mechanism and models may emerge, for example electroluminescence triggered by triboelectricity was proposed for the peculiar ML behaviour of ZnS:Cu${}^{+}$ [122,334], and they are supposed to reveal common features of ML. Nevertheless, the role of defects is essential. Considering the connection with the phenomenon of persistent luminescence, the trap density and trap depth distribution is crucial for the ML phenomenon. Having more defect sites to trap charge carriers implies a potentially higher ML intensity, e.g., the non-stoichiometry in ZnS:Cu increases ML via sulfur vacancies [335]. Shallow traps, however, are often thermally detrapped before the start of the ML experiment. Deeper traps can also be helpful for the reasons that ML can be found when persistent luminescence has dropped to very low level, or their transition levels of defects match well with that of emitting centres, or they interact with shallow traps. The interplay between these levels under dynamical loads also opens the door to tune ML properties. For example, the shallow and deep traps in ZnS:Cu can be active under different frequency of loads, leading to a change of emission spectrum [121,123]. Point defects and extended defects are valuable in ML phosphors, especially whose host is piezoelectric, in that they may create large internal electric fields and thus reduce detrapping barriers. In a typical ML experiment where stress is around several tens of MPa, a dramatic change of the band gap of the host is not likely, which means internal electric fields caused by defects play a role in detrapping of carriers. A reduction of the detrapping barrier can be achieved by a change of the structure of defects which is so flexible that even small magnitude of stress induces appreciable shift of the detrapping barrier. This presumably explains why ML is mainly found in compounds with flexible framework structures. Additionally, extended defects, such as dislocations and domain walls, induce changes in the electronic structure of ML phosphors since they tend to move under certain mechanical loads. It is clear that a better understanding of ML will only be achieved after a deep investigation of defects in ML phosphors.

The modelling of the dynamics of ML at various time scales is also vital for the purpose of comparing simulations with experiments. This is the way to connect ML to the microscopic feature of ML phosphors, while thermodynamics (often non-equilibrium thermodynamics) can make sense for the irreversible nature of ML process, maybe even creating new research tracks in ML.

## 5. Proven and Potential Applications

ML materials can emit light under mechanical stress of various forms. These materials can thus have a large number of applications in stress sensing, dynamic pressure mapping, light sources or the detection of electric and magnetic fields. The key performance of these sensors relies on the properties of the ML process, especially the sensitivity, the strain-luminescence response and the self-recovery effect (durability). In the case of mapping stress distributions, the most desirable indicator for ML phosphors is a small threshold and a good linearity. A small threshold stress value will extend the range of mapping to very low stress levels while good linearity between ML and stress provides very reliable values of the amount of stress in a large range. For dynamic mapping of stress, the contrast for different types of stress is of great importance and a large sensitivity is thus desired. Meanwhile, the maximum stress where linearity holds is also important for cases with local concentration of stress. Below, the key application areas of ML materials are outlined.

### 5.1. Visualization of Stress Distribution

Mapping stress distributions when stress is applied or monitoring total stress of a component under performance, is extremely informative when studying repetitive loading of actual components or in the design phase of structural components. Based on the linear relationship between stress and instantaneous ML intensity, ML phosphors provide an alternative choice for other sensor materials because of their flexibility, limited invasiveness and easy adjustability when compared to electric or fibre-based strain gauges. These ML materials can be incorporated in a translucent matrix, or painted on the surfaces of interested objects or embedded in other composites as long as collecting ML intensity is accessible and convenient.

**Figure 17:**
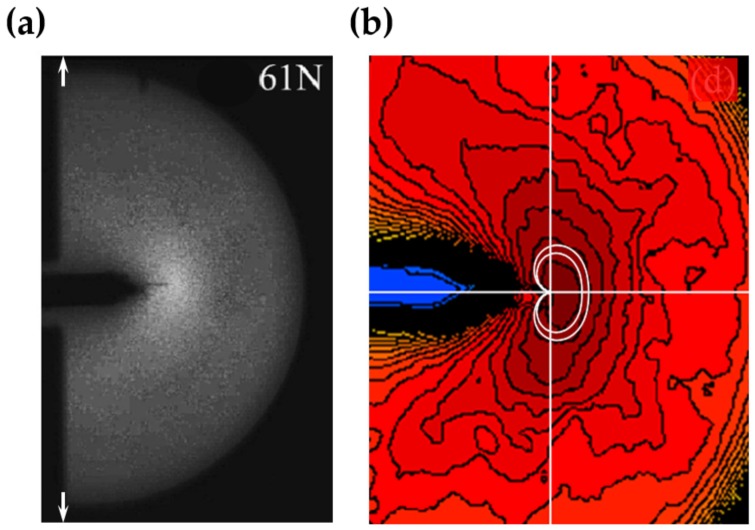
Digital Image of ML (**a**) in the vicinity of the crack tip of a compact tension specimen under a load of 61 N (rate: 80 N/s) where white arrows show the load manner, and its comparison (**b**) (isostress contour lines (black)) with theoretical calculations (white line). Reproduced from Ref [19], Copyright (2013), with permission from Elsevier.

The stress intensity factor (SIF) *K* is used in the fracture mechanism to predict the stress state near the tip of the crack by a remote load. In the first mode (pure opening mode), crack surfaces move directly apart and the SIF is denoted by $K_{I}$. Timilsina et al. have explored the possibility of calculating $K_{I}$ from the emission of ML materials coated on stainless steel under compact tension [19]. The instantaneous ML intensity is shown in Figure 17a, and contours were constructed in Figure 17b accordingly. The ML intensity was treated as a stress distribution from elasticity relations, and thus $K_{I}$ can be calculated from data points on these contour lines. Based on the assumption that the stress deviator is responsible for the ML, the calculated $K_{I}$ agrees well with values measured according to methods proposed by American Society for Testing of Materials. Similarly, the plastic SIF can be even calculated from the accumulated ML intensity from the plastic field in the vicinity of a crack [336]. It extends ML into the field of complex nonlinear plasticity under complex loading conditions.

Further research validated the possibility of measuring full field strain (Von Mises strain) via ML phosphors [292]. The visualization of propagation of cracks was realized by Kim et al. [90]. The stress distribution of the composite (containing 30 wt % SrAl${}_{2}$O${}_{4}$:Eu${}^{2+}$) was modeled by solving the dynamic stress field under mixed-mode conditions. The theoretical calculation agrees well with the stress distribution contour obtained from the ML intensity [20]. With the help of a high speed camera (8000 fps), the quasi-dynamic materials resistance curve (*R*-curve) of zirconia polycrystals can be measured for crack speeds up to 15 m/s [18]. This method illustrates the feasibility of visualizing and calculating static stress field from ML materials and can even be extended to dynamic conditions.

**Figure 18:**
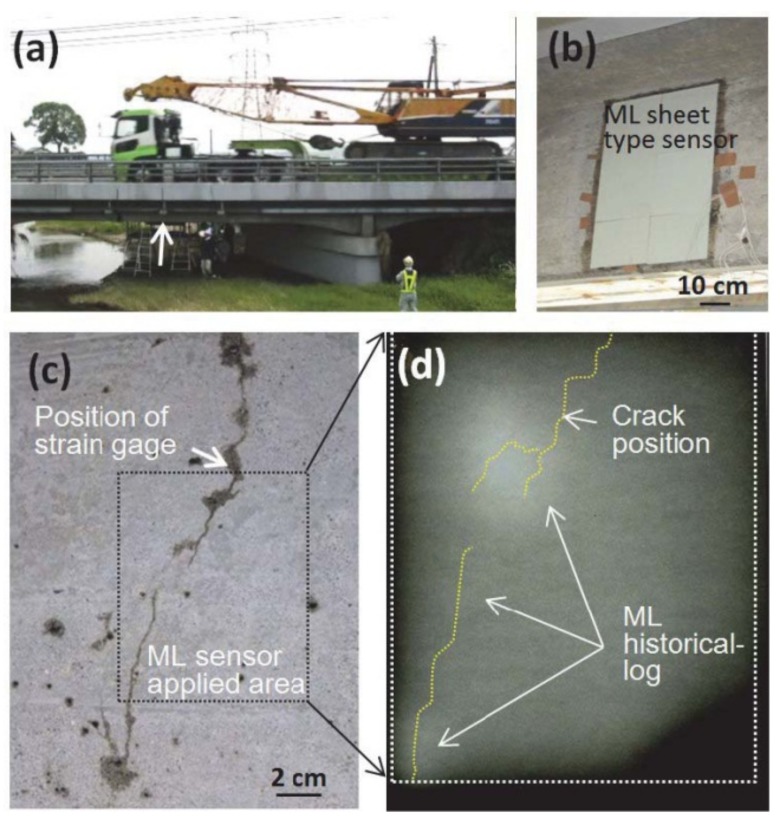
Health monitoring at a bridge in use with full view (**a**) by attaching ML sheet type sensor (**b**) to the original concrete girder surface (**c**) (dotted line), and the ML integral image using the historical-log recording system (**d**). © 2013 IEEE. Reprinted, with permission from Ref [337].

Dynamic stress sensing is also crucial for the diagnosis of possible damage in constructions via a structural health monitoring process. Recently, Xu et al. demonstrated the possibility of applying ML paint as a historical-log stress system for monitoring crack evolution on a bridge in use under general traffic conditions [337] (Figure 18a,b). The ML particles SrAl${}_{2}$O${}_{4}$:Eu${}^{2+}$ were mixed with epoxy resin which is then coated on the bridge. Another photoelectric layer is put on the ML sheet with black packing tape to detect the light intensity that was emitted from the ML materials (Figure 18c, dotted area). When vehicles passed along the bridge, the stress in the concrete increases and ML emission was recorded by the photosensitive layer. The overlap of the position of a visible crack and the recorded ML intensity (Figure 18d) indicates that ML paint can indeed be used as historical-log recording system. Such function of monitoring fatigue cracks via ML has been demonstrated in cases such as steel vessels [338] and hydrogen tanks [339].

**Figure 19:**
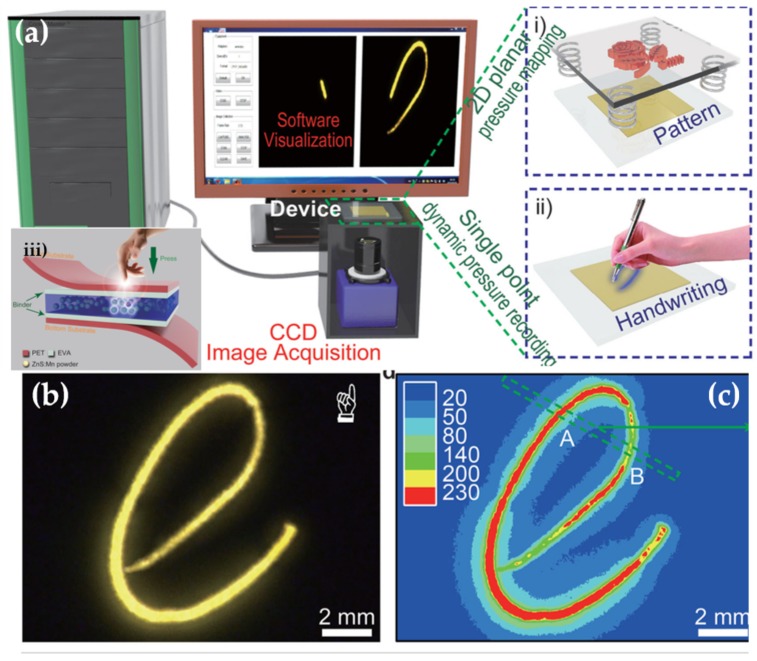
The schematic illustration of the image acquisition and processing system (**a**) (schematic structure of ZnS composite film in **iii)**), which is capable of mapping 2D planar pressure **i)** and detecting single-point dynamic pressure **ii)**, and the digital visualization (**b**) and its pressure distribution contour (**c**) of a handwritten “e” via ML method. Reproduced from Ref [108], with permission from John Wiley and Sons.

Wang et al. [108] recently devised a signature recording system thanks to the self-recovery of ZnS:Mn${}^{2+}$. The schematic components of the system are depicted in Figure 19a. A signature of letter “e” was recorded by digital imaging (Figure 19b) and its corresponding pressure distribution can be extracted via ML intensity (Figure 19c). This technique would give signatures another merit, i.e., pressure mapping, which makes them quite personalized. In a complicated system, the limited sensitivity of ZnS:Mn${}^{2+}$ at low pressure range can be compensated by triboelectric sensor layer, which enables one to sense dynamic pressure range in our daily life [340].

### 5.2. Visualization of Ultrasonic Fields

**Figure 20:**
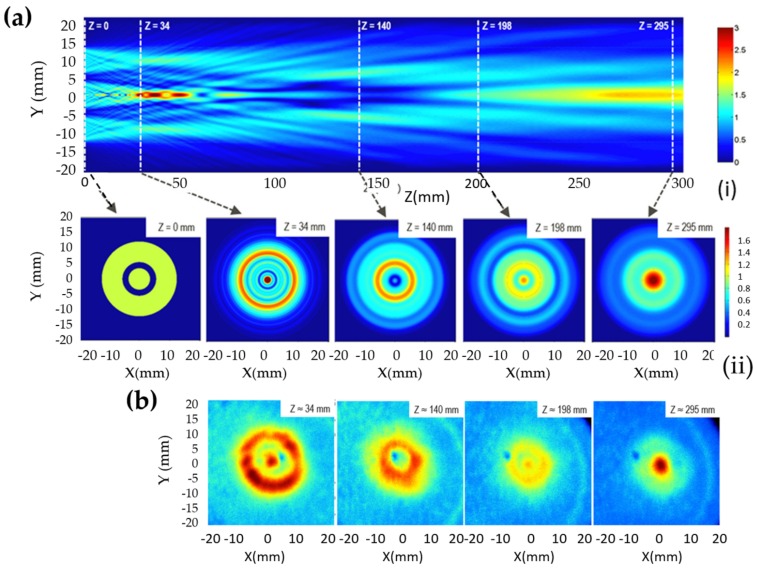
The simulation (**a**) of the ultrasound intensity at different distances from the source (beam transducer), and the ML intensity distribution (**b**) of the BaSi${}_{2}$O${}_{2}$N${}_{2}$:Eu sensing material. Reproduced from Ref [16], with the permission of AIP Publishing.

Ultrasound produces pressure at the wavefront of sound waves, and some ML materials are able to show luminescence upon stimulation of ultrasound. Terasaki et al. demonstrated the feasibility of ML due to ultrasound radiation to act as light sources in vivo or as light source for photo-catalysis [341,342]. However, the pressure field produced by an ultrasound wave was only visualized in detail with the help of BaSi${}_{2}$O${}_{2}$N${}_{2}$:Eu${}^{2+}$ by Kersemans et al. in 2015 [16]. BaSi${}_{2}$O${}_{2}$N${}_{2}$:Eu${}^{2+}$ is able to emit light which is proportional to the power of the ultrasound produced by a transducer. Thus, an epoxy plate was placed at different distances to the transducer to sense pressure via ultrasound induced light emission. ML intensities were first recorded by a camera and the data were then retrieved digitally from software to represent the pressure distribution at the corresponding distances (Figure 20b). The pressure distribution obtained via this method was compared to the one obtained by directly scanning the interested area via a hydrophone. A good correlation between the two methods has been found, and the agreement is supported by simulations (Figure 20a). Therefore, it is possible for researchers to reconstruct 3D ultrasonic fields through ML materials in a fast and efficient manner.

### 5.3. Light Sources and Displays

**Figure 21:**
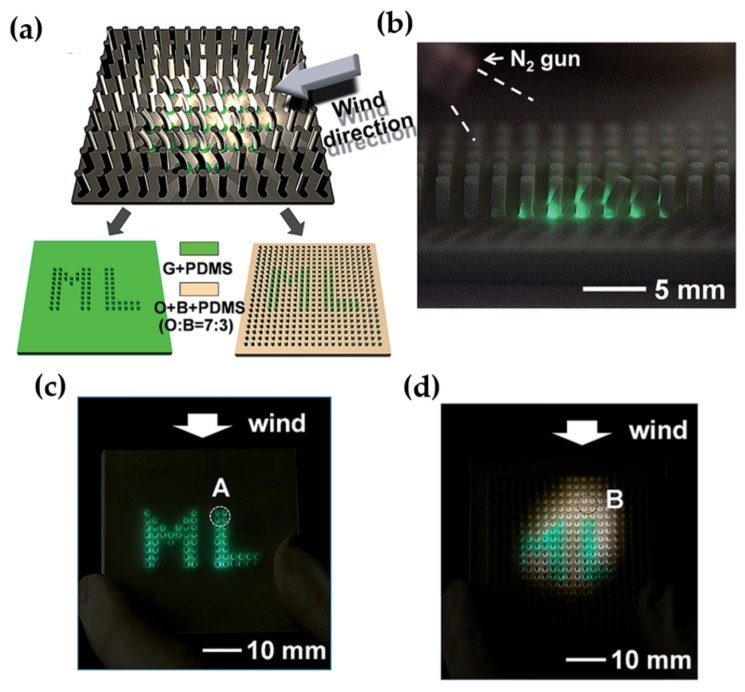
Schematic illustration of set-up (**a**) for wind-driven ML display, and photographs showing the emission form ML phosphors upon N${}_{2}$ flow (**b**) and images of “ML” letter in different background colours: dark (**c**) and yellowish (**d**) due to different fabrication structures. Reproduced from Ref [17] Published by The Royal Society of Chemistry.

Mechanoluminescent materials in general can be viewed as energy storage materials that are able to release energy (and thus light) upon mechanical stimulus. Intuitively, the emission can be used as a light source under proper kind of stimulation from the environment, for example wind, gas turbulence, or friction. Jeong et al. has taken advantage of the self-recovery of ZnS phosphors doped with Mn${}^{2+}$ or Cu${}^{+}$. ZnS:Cu${}^{+}$ in PDMS films maintained at least 80% of its original intensity as it underwent 30 thousands cycles of stretching and releasing [121]. Later on, they discovered that ZnS doped with Mn${}^{2+}$ or Cu${}^{+}$ could emit light when they are subjected to the force of wind [17] (Figure 21a). As shown in Figure 21b–d, the micro-pillar coated with ML particles could emit light when exposed to a N${}_{2}$ gun and the “ML” image can be clearly seen (Figure 21b,c). The light output is proportional to the gas flow rate, and by mixing of ZnS:Mn${}^{2+}$ and ZnS:Cu${}^{+}$, various colour temperatures can be generated [127] (Figure 21d). Unfortunately, the efficiency of this process was not reported, which may impose limits on the application of ML phosphors as display materials in cases where wind is the only excitation source. Meanwhile, the tuning of emission spectra can be achieved by mixing different ratio of ZnS:Mn/ZnS:Cu [127] or using dyes as spectrum converter based on energy transfer [126].

### 5.4. Sensing Other Fields

**Figure 22:**
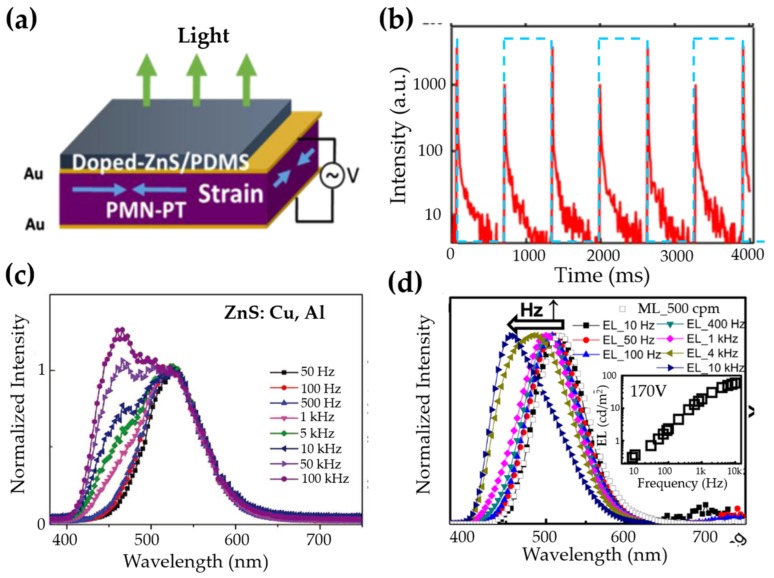
The structure of the piezo-phototronic luminescence device (**a**) and its luminescence intensity λ= 508 nm (**b**) under a square-wave voltage (blue dashed line) stimulus (Reproduced from Ref [13], Copyright (2015), with permission from Elsevier); the change of emission spectrum with increasing electric field of ZnS:Cu${}^{+}$, Al${}^{3+}$ films (**c**) (Ref [123], with permission from John Wiley and Sons), and of ZnS:Cu${}^{+}$ phosphor (**d**) (Ref [121], with the permission of AIP Publishing).

It is not surprising that ML materials can sense strains induced from other types of fields. Zhang et al. showed that ZnS:Mn${}^{2+}$ films deposited on a PMN-PT single crystal can emit light when an AC electric field was applied to the crystal [117]. The strain experienced by the coated ML films could result in light emission, with the output power [123] and emission spectra being tunable by the frequency of electric field in the case of ZnS:Cu${}^{+}$ (Figure 22a–d) [13].

**Figure 23:**
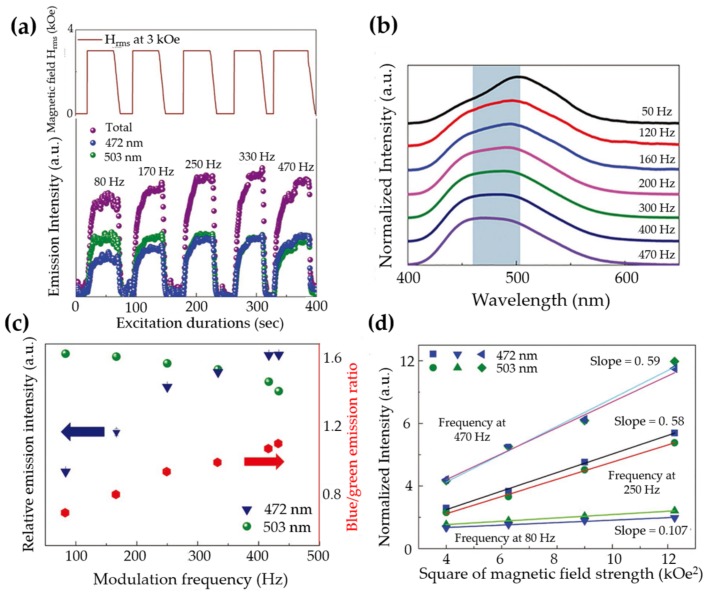
The time dependent luminescence profiles (**a**); the emission profile (**b**); and the normalized emission intensity (**c**) of ZnS:Cu${}^{+}$, Al${}^{3+}$ emitting at 472 and 503 nm under a sinusoidal magnetic field with various frequencies and the dependency on the square of the magnetic-field strength (**d**) with the corresponding linear fittings. Reproduced from Ref [125], with permission from John Wiley and Sons.

Besides, Wong et al. demonstrated that ZnS:Al${}^{3+}$, Cu${}^{+}$ phosphors could yield light emission under a magnetic field when they are mixed with Fe-Co-Ni alloys and PDMS [22]. The ML intensity could indicate the application and removal of the AC magnetic field (Figure 23a–d), while the intensity was proportional to the frequency and the square of the magnetic field intensity. Tuning of the emission can also be obtained by tuning the frequency of the alternating magnetic field [125]. Such a new type of device is capable of sensing or converting a dynamic magnetic action without the need of using a power unit for the device.

These two examples also demonstrate that ML particles, especially piezoelectric ones, could respond to various fields that cause strain by emission of light.

### 5.5. Design Consideration for ML Phosphors

Stresses and strains are so diverse that sensing stress fields can be of interest from a practical view. The application of ML phosphors in sensing stress relies more on the behaviour of ML phosphors than on the precise knowledge of the release mechanisms of carriers. It is possible to improve the desired merit of properties, such as efficiency, sensitivity and the range of linearity, by alloying cations or anions, codoping or tuning syntheses parameters of known ML phosphors. It demands understanding of the ML mechanism and also imagination to discover new host and dopants combinations in practice, but researchers do have some options. Searching derivatives of known ML hosts may provide more chances to find novel hosts (Notice that most of ML hosts can be grouped in a certain type of structure as in Table 1), since similar hosts provide similar structure chemistry and elastic properties. Compounds consisting of layers or flexible framework (which is the case for most ML hosts), which in addition show photoluminescence or persistent luminescence, open the door for appreciable change of defects for ML. Phase transitions, especially Martensic phase transitions, can favour strong ML because of the presence of various kinds of microstructures produced in these transitions. With the help of these considerations, researchers may draft their own candidates of ML host and screen them via doping with metal impurities. Unexpected outcomes are in turn helpful for the study of ML mechanism. With more proper ML phosphors and a better understanding of ML mechanism, we can also expect the application of ML in other fields, such as bio-imaging using ultrasound, and wearable devices for safety signalling.

## 6. Conclusions and Outlooks

Despite the promising application potential of mechanoluminescence, the research field is still faced with difficulties in understanding the mechanism of ML and in developing novel ML materials. A thorough survey of crystal structures of ML phosphors shows the complexity posed by the co-existence of anisotropy of chemical bonding and certain types of microstructure such as twins, domains and modulations. The guidelines for searching ML candidates are thus more complicated than just identifying piezoelectric persistent phosphors with elastic anisotropy, an implicit hint gained from a literature study of proposed mechanisms and reported compounds. The emission wavelength and its tunability is also very limited. For example, there is a severe lack of ML phosphors that emit near infra-red light, which can be fascinating in biomedical imaging using ultrasound radiation. Challenges come from a complex interaction of anionic frameworks and microstructures with dopants, depending on the specific material under investigation as well. The change of energy levels of dopants upon stress is also unclear even for the most studied ML materials. The lack of the much desired solid evidence keeps the study of the mechanism behind ML on the level of phenomenological modelling. In this sense, in depth studies of how defect levels change under various strain at the atomic level is highly desired. Density functional theory could soon be up to this task, due to an ever improved precision in predicting the position of impurity levels in the band gap with the help of hybrid functionals, GW method, or other methods with improved accuracy. The release of carriers can be related to the intrinsic defects of ML materials or related to extended defects, which has been evidenced by several research on the mechanism, as discussed in Section 4. An important improvement in the mechanism of ML is the recognition of the hysteresis of the ML itself.The relevant model is not only suitable to predict instantaneous ML intensity under varying load conditions, but also successful in explaining the presence of a ML peak upon abruptly releasing the mechanical load. Hysteresis in piezoelectricity, ferroelasticity and ferromagnetism are based on the movement of domain states. Obviously, ML is not reversible under strains and thus the existence of “domain states” in ML phenomenon are not yet available. This provides a good direction for future research.

Unexpected outcomes from efforts of discovering novel ML phosphors in turn can reveal insights in the mechanism behind ML. A well-known example is the relation of Sr${}^{2+}$ ordering in the Ca${}_{1-x}$Sr${}_{x}$Al${}_{2}$Si${}_{2}$O${}_{8}$ series, which causes the absence of ML at the morphotropic transition point. Phase transitions due to temperature or chemical substitution can thus be used to tune the properties of ML, the reason being that phase transitions may lead to lattice distortion, cation or anion orderings or domain states which are apparently important for ML. Given that a large proportion of ML compounds show certain types of phase transitions (Table 2), a promising research direction would be the influence of phase transition on elastic properties, electronic properties and ML performances of these compounds. Equally importantly, a standardized procedures to characterize ML phosphors would make comparison of different ML phosphors possible and thus lower the difficulties of discovering novel materials.

Despite the challenges ahead, the development of ML can be enhanced by our deeper understanding of the ML mechanism, by our efforts in tuning ML properties and by carefully analysing the trial-and-error synthesis attempts. The intriguing mechanisms and promising applications deserve an intense research in this particular field of luminescence, which requires efforts from chemists, physicists and engineers.

## Figures and Tables

**Table 1:** Reported mechanoluminescent compounds and their properties.

Structure	Host	Space Group	Piezo ${}^{⋄}$	Dopants ${}^{\Delta }$	$\lambda_{ML}$ (nm)	PerL, Trap Depth (eV) ${}^{▽}$	Load ${}^{★}$	ML Indicators ${}^{†}$
Rock Salt	KCl	Fm$\bar{3}$m	−	-	430, 520	+ [63,64], *0.22*, 0.37, 0.51, 1.12 [65]	E, P [63,64,66,67]	?, ?
	KCl	Fm$\bar{3}$m	−	Eu${}^{2+}$	420	+ [68]	E, P [69]	?, ?
	KI	Fm$\bar{3}$m	−	-	515	+ [63,64], 0.20, 0.21, 0.22, *0.29*, 0.32, 0.43 [70]	E, P [63,64]	?, ?
	KBr	Fm$\bar{3}$m	−	-	465	+ [63,64], *0.27*, 0.28, 0.29, 0.35, 0.36, 0.44 [71]	E, P [63,64]	?, ?
	NaF	Fm$\bar{3}$m	−	-	270, 306, 334, 410, 610	+ [64], *0.31*, 0.57, 0.67, 0.84, 1.0, 1.22 [70]	E, P [64]	?, ?
	NaCl	Fm$\bar{3}$m	−	-	282, 318, 362, 610	+ [63,64], 0.74, *0.86*, 0.99, 1.20, *1.47* [71]	E, P [63,64]	?, ?
	LiF	Fm$\bar{3}$m	−	-	290, 560, 650	+, 0.25, 0.26, 0.36, 0.46, 0.53, 0.78 [70]	E, P [63,64,72]	
	RbCl	Fm$\bar{3}$m	−	-	510, 430	+ [64], 1.65 [73]	E, P [64]	?, ?
	RbBr	Fm$\bar{3}$m	−	-	465, 495	+ [64], 1.55, 1.78 [74]	E, P [64]	?, ?
	RbI	Fm$\bar{3}$m	−	-	525, 630	+ [64], ?	E, P [64]	?, ?
	MgO	Fm$\bar{3}$m	−	-	420, 520	?, 0.31 [75]	E, P [76]	(0.1, ?), ?
Tridymite	SrAl${}_{2}$O${}_{4}$	P2${}_{1}$	+	Eu${}^{2+}$	520	+, 0.77 [77,78]	E, T [79]	(40, 900) [19], -
	SrAl${}_{2}$O${}_{4}$	P2${}_{1}$	+	Eu${}^{2+}$/Dy${}^{3+}$	520	+, 0.65 [77,78]	E [80,81,82]	(0, >1.0 k), -
	SrAl${}_{2}$O${}_{4}$	P2${}_{1}$	+	Eu${}^{2+}$/Er${}^{3+}$	524, 1530	+ [77,78], 0.75 [83]	E [84]	(30, 750)(IR), -
	SrAl${}_{2}$O${}_{4}$	P2${}_{1}$	+	Ce${}^{3+}$	381, 450	+ [85], 0.75 [86]	E [87]	(∼0, 500), 0.005
	SrAl${}_{2}$O${}_{4}$	P2${}_{1}$	+	Ce${}^{3+}$/Ho${}^{3+}$	381, 450	+ [85], ?	E [87]	(30, 700), 0.052
	SrAl${}_{2}$O${}_{4}$	P2${}_{1}$	+	Eu${}^{2+}$/Dy${}^{3+}$/Nd${}^{3+}$	520	+ [88] , ?	E, P [89,90]	-, -
	SrAl${}_{2}$O${}_{4}$	P2${}_{1}$	+	Eu${}^{2+}$/B${}^{3+}$	520	+, 0.77	E, T	?, -
	CaAl${}_{2}$O${}_{4}$	P2${}_{1}$/n	−	Eu${}^{2+}$	440	+ [91], 0.42, 0.53, 0.58 [92]	I [93]	-, -
	CaAl${}_{2}$O${}_{4}$	P2${}_{1}$/n	−	Tb${}^{3+}$	495,545	+, 0.66, 1.08 [94]	I [95]	-, -
	Sr${}_{1-x}$Ba${}_{x}$Al${}_{2}$O${}_{4}$ (*x* = 0, 0.1, 0.2, 0.4)	P6${}_{3}$(*x* = 0.4) P2${}_{1}$(others)	+	Dy${}^{3+}$	480,575	?, ?	T [96]	-, -
	Sr${}_{1-x}$Ba${}_{x}$Al${}_{2}$O${}_{4}$ (*x* = 0, 0.1, 0.2, 0.4)	P6${}_{3}$(*x* = 0.4) P2${}_{1}$(others)	+	Eu${}^{2+}$/Eu${}^{3+}$	520, 613, 700	+ [97], ?	T [98]	-, -
	Sr${}_{0.9}$Ca${}_{0.1}$Al${}_{2}$O${}_{4}$	P2${}_{1}$	+	Dy${}^{3+}$	480, 575	+, ?	T [96]	-, -
	Zn${}_{2}$Ge${}_{0.9}$Si${}_{0.1}$O${}_{4}$	R3	+	Mn${}^{2+}$	535	+, 40 ${}^{\circ }$C [99]	E [100]	(600, 2.8k), ∼10${}^{-3}$
Spinels	MgGa${}_{2}$O${}_{4}$	Fd$\bar{3}$m	−	Mn${}^{2+}$	506	+, 60 ${}^{\circ }$C [101]	T [101]	?, ?
	ZnGa${}_{2}$O${}_{4}$	Fd$\bar{3}$m	−	Mn${}^{2+}$	505	+, 70 ${}^{\circ }$C [102]	T [101]	?, ?
	ZnAl${}_{2}$O${}_{4}$	Fd$\bar{3}$m	−	Mn${}^{2+}$	560	+, 125 ${}^{\circ }$C [103]	T, I [103,104,105]	?, ?
Wurtzite	ZnS	P6${}_{3}$mc	+	Mn${}^{2+}$	585	−, 0.3 [106]	E, P, T [107,108,109,110,111,112,113,114,115,116,117]	(*0.6*, *50*), *0.7*|*2.2*
	ZnS	P6${}_{3}$mc	+	Cu${}^{+}$	520	+ [118], 0.20–0.30, 0.42–0.46, 0.54–0.60 [119,120]	E [14,121,122,123,124,125,126]	?, ?
	ZnS	P6${}_{3}$mc	+	Mn${}^{2+}$/Cu${}^{+}$(Al${}^{3+}$)	517, 587	+, ?	E [13,17,22,127,128]	?, ?
	ZnS	P6${}_{3}$mc	+	Cu${}^{+}$/Al${}^{3+}$	(475), 525	+, ?	E [22,123]	?, ?
	ZnTe	P6${}_{3}$mc	+	Mn${}^{2+}$	?	−, ?	T [129]	∼0, ?
	(ZnS)${}_{1-x}$(MnTe)${}_{x}$ (*x* < 1/4)	P6${}_{3}$mc	+	Mn${}^{2+}$	650	?, ?	I [130,131,132]	?, ?
	CaZnOS	P6${}_{3}$mc	+	-	438, 515, 614	−, 0.17, 0.58 [133]	T, E, I [133]	?, ?
	CaZnOS	P6${}_{3}$mc	+	Mn${}^{2+}$	580	−, *0.18*, 0.58, 0.67 [134]	T, E [134,135]	(0, 1.0 k), ∼0.325
	CaZnOS	P6${}_{3}$mc	+	Cu${}^{+}$	450, 530	+, 0.42, 0.71 [136]	E [136,137,138]	(0, 350), ?
	CaZnOS	P6${}_{3}$mc	+	Er${}^{3+}$	525, 540, 560	− [139], ?	E [139]	?, ?
	CaZnOS	P6${}_{3}$mc	+	Sm${}^{3+}$	566, 618, 655	−, ?	E, T [140]	(*13*, *65*), *0.48*
	BaZnOS	Cmcm	−	Mn${}^{2+}$	634	+ [141], 0.83 [142]	E [141,142]	(∼100, 5.0 k), 1.0
Melilite	Ca${}_{2}$MgSi${}_{2}$O${}_{7}$	P$\bar{4}$2${}_{1}$m	+	Eu${}^{2+}$	530	+ [143], 90 ${}^{\circ }$C [144]	E [145]	(0, 1.0 k), <$10^{-3}$
	Ca${}_{2}$MgSi${}_{2}$O${}_{7}$	P$\bar{4}$2${}_{1}$m	+	Eu${}^{2+}$/Dy${}^{3+}$	530	+, 0.66 [143,146,147]	E [148,149,150]	(∼70, 1.0 k), 1.433
	Sr${}_{2}$MgSi${}_{2}$O${}_{7}$	P$\bar{4}$2${}_{1}$m	+	Eu${}^{2+}$	499	+ [151], 0.50 [11]	E [11]	(∼70, ?), 1.134
	Sr${}_{2}$MgSi${}_{2}$O${}_{7}$	P$\bar{4}$2${}_{1}$m	+	Eu${}^{2+}$/Dy${}^{3+}$	460	+, 0.75 [147,152,153]	I [148,154]	?, ?
	Ba${}_{2}$MgSi${}_{2}$O${}_{7}$	C2/c	+	Eu${}^{2+}$/Dy${}^{3+}$	495	+ [155], ?	I [156]	?, ?
	SrCaMgSi${}_{2}$O${}_{7}$	P$\bar{4}$2${}_{1}$m	+	Eu${}^{2+}$	496	+, 0.47 [11]	E [157]	(∼70, 1.0 k), 1.02
	SrBaMgSi${}_{2}$O${}_{7}$	P$\bar{4}$2${}_{1}$m	+	Eu${}^{2+}$	440	+, 0.55 [11]	E [11]	(∼0, 1.0 k), 0.213
	Sr${}_{n}$MgSi${}_{2}$O${}_{5+n}$ (1≤n≤2)	P$\bar{4}$2${}_{1}$m	+	Eu${}^{2+}$	468	+, 0.45, 0.78 [158]	E [158]	?, ?
	Ca${}_{2}$Al${}_{2}$SiO${}_{7}$	P$\bar{4}$2${}_{1}$m	+	Ce${}^{3+}$	417	+, 90 ${}^{\circ }$C [159]	E [23,160]	(220, 1.0 k), -
	Sr${}_{2}$Al${}_{2}$SiO${}_{7}$	P$\bar{4}$2${}_{1}$m	+	Eu${}^{2+}$	484	+, 0.88 [161]	I [162,163]	-, -
	Sr${}_{2}$Al${}_{2}$SiO${}_{7}$	P$\bar{4}$2${}_{1}$m	+	Eu${}^{2+}$/Dy${}^{3+}$	484	+, 0.52 [161]	I [162,163]	-, -
	CaYAl${}_{3}$O${}_{7}$	P$\bar{4}$2${}_{1}$m	+	Eu${}^{2+}$	440	+ [164], ?	E [165]	(30, 1.0 k), 0.03
	CaYAl${}_{3}$O${}_{7}$	P$\bar{4}$2${}_{1}$m	+	Ce${}^{3+}$	425	+, 80 ${}^{\circ }$C [159]	E [166]	(10, 1.0 k), -
Anorthite	CaAl${}_{2}$Si${}_{2}$O${}_{8}$	P$\bar{1}$	−	Eu${}^{2+}$	430	+, 0.68, 0.82 [167]	E [168]	(33, >1.0 k), 0.748
	Ca${}_{1-x}$Sr${}_{x}$Al${}_{2}$Si${}_{2}$O${}_{8}$ (x<0.8)	I$\bar{1}$	−	Eu${}^{2+}$	406–440	+ [169], ?	E [168,170,171]	(44, 400), 1.12 (*x* = 0.4)
	SrMg${}_{2}$(PO${}_{4}$)${}_{2}$	P$\bar{3}$m	+	Eu${}^{2+}$	412	+, 0.77 [172]	E [57]	(∼0, >1.0 k), -
Perovskite	Ba${}_{1-x}$Ca${}_{x}$TiO${}_{3}$ (0.25<x<0.8)	P4mm+Pbnm	+	Pr${}^{3+}$	612	+ [173], 0.356 [174]	E [175,176,177,178]	(70, >1.0 k), 0.006
	Ba${}_{1-x}$Ca${}_{x}$TiO${}_{3}$	P4mm+Pbnm	+	Pr${}^{3+}$/Dy${}^{3+}$	612	+, 0.35 [173]	E [174,179]	(∼0, >900), 0.003
	Ba${}_{1-x}$Ca${}_{x}$TiO${}_{3}$	P4mm+Pbnm	+	Pr${}^{3+}$/La${}^{3+}$-Lu${}^{3+}$	612	+, 0.31–0.38 [173], 0.00 for Ce${}^{3+}$ [173]	E [174]	?, ?
	LiNbO${}_{3}$	R3c	+	Pr${}^{3+}$	612	?, ?	E [180]	(30, 1.0 k)?, -
	Sr${}_{2}$SnO${}_{4}$	Pccn	−	Sm${}^{3+}$	575, 625, 660	+ [181], 117 ${}^{\circ }$C, 187 ${}^{\circ }$C [182]	E [183]	(15, >250), -
	(Ca, Sr, Ba)${}_{2}$SnO${}_{4}$	Pccn	−	Sm${}^{3+}$/La${}^{3+}$	575, 625, 660	+, *0.56*, 0.84, 1.35 [185,186]	E [186]	(20, 100), -
	Sr${}_{3}$Sn${}_{2}$O${}_{7}$	Amam	−	Sm${}^{3+}$	575, 625, 660	+ [184], ?	E [183]	(15, 250)?, -
	Sr${}_{3}$(Sn, Si)${}_{2}$O${}_{7}$	Amam	−	Sm${}^{3+}$	575, 625, 660	+, 113 ${}^{\circ }$C [187]	E [187]	(?, ∼2350), ∼1.3
	Sr${}_{3}$(Sn, Ge)${}_{2}$O${}_{7}$	Amam	−	Sm${}^{3+}$	575, 625, 660	+, 117 ${}^{\circ }$C [187]	E [187]	(?, ∼2350), ∼2.1
	Ca${}_{3}$Ti${}_{2}$O${}_{7}$	Ccm2${}_{1}$	+	Pr${}^{3+}$	615	+, 0.69–0.92 [188]	E,T [189]	(∼10, 200), -
	CaNb${}_{2}$O${}_{6}$	Pbcn	−	Pr${}^{3+}$	613	− [29], 0.71, 0.81, 1.04 [29]	E [29]	(∼30, 1.0 k), 0.001
	Ca${}_{2}$Nb${}_{2}$O${}_{7}$	P2${}_{1}$	+	Pr${}^{3+}$	613	+ [29], 0.60, *0.89*, *1.04*, 1.27 [29]	E [29]	(∼0, 1.0 k), 0.016
	Ca${}_{3}$Nb${}_{2}$O${}_{8}$	Fm3m or P4/nnc	+	Pr${}^{3+}$	613	− [29], 0.74 [29]	E [29]	(∼50, 1.0 k), 0.004
MSi${}_{2}$O${}_{2}$N${}_{2}$ (M = Ca, Sr, Ba)	BaSi${}_{2}$O${}_{2}$N${}_{2}$	Cmcm	+	Eu${}^{2+}$	498	+ [190], 0.46, 0.48 [191]	E [192]	-, -
	SrSi${}_{2}$O${}_{2}$N${}_{2}$	P1	+	Eu${}^{2+}$	498	+, 100 ${}^{\circ }$C [190]	E [192]	-, -
Others	CaZr(PO${}_{4}$)${}_{2}$	Pna2${}_{1}$ [193]	+	Eu${}^{2+}$	474	+, *0.69*, 0.83, 1.17, 1.87 [194]	E [194]	(5, >2.0 k), -
	ZrO${}_{2}$	P2${}_{1}$/c	−	Ti${}^{4+}$	470	+ [195], 0.81, *1.00*, 1.16 [196]	E [197,198]	(∼0, 100), -

**Notes**: Piezo ${}^{⋄}$: + = piezoelectric, − = not piezoelectric; Dopants ${}^{\Delta }$: − = undoped; PerL, trap depth (eV) ${}^{▽}$: PerL = Persistent luminescence, + = yes, − = no, the main traps are in italic; Load ${}^{★}$: E = Elastic, P = Plastic, T = Friction, I = low velocity impact; ML Indicators ${}^{†}$: format: linear range (N${}_{min}$, N${}_{max}$) in Newton (italic for MPa), sensitivity in kcps/N (italic for kcps/MPa). ? denote unknown and - incalculable from the references.

**Table 2:** Structure disorder at the microstructure level for some ML compounds.

Host	HT Phase ${}^{⋄}$	Atomic Order ${}^{♡}$	Domains ${}^{▹}$	Twins
SrAl${}_{2}$O${}_{4}$	P6${}_{3}$ (680–860 ${}^{\circ }$C) P6${}_{3}$22 (>860 ${}^{\circ }$C)	Al${}^{3+}$ displacement, modulation q = (0, 0.5, 0) [30,247,248]	translational, ferroelastic, ferroelectric [30,247,248]	3 types [30,247,248]
BaAl${}_{2}$O${}_{4}$	P6${}_{3}$22 [249,250,251]	modulation q = (0.5, 0, 0) (<250 K) [252]	translational, orientational, nano, APBs [249,250]	180${}^{\circ }$ rotational twins [249,250]
Sr${}_{0.9}$Ca${}_{0.1}$Al${}_{2}$O${}_{4}$	P6${}_{3}$22, C2 [253]	?	ferroelectric	?
Sr${}_{1-x}$Ba${}_{x}$Al${}_{2}$O${}_{4}$ (0≤x≤0.3)	P6${}_{3}$22, C2 [253]	Al${}^{3+}$ displacement, modulation q = (0, 0.5, 0) [253]	ferroelastic ferroelectric	twins [247]
Sr${}_{0.6}$Ba${}_{0.4}$Al${}_{2}$O${}_{4}$	P6${}_{3}$22 (>500 K) [214]	[AlO${}_{4}$] fluctuation, O disorder [253,254]	ferroelectric [253,254]	?
MgGa${}_{2}$O${}_{4}$	?	Mg disorder, inverse spinel [101]	?	?
CaAl${}_{2}$Si${}_{2}$O${}_{8}$	I$\bar{1}$ [255] (>514 K)	∼8% Al, Si disorder [256]	APBs [257]	twins [258]//(010)
SrAl${}_{2}$Si${}_{2}$O${}_{8}$	Immm [259]	Al, Si disorder	APBs [259]	twins [259]
BaAl${}_{2}$Si${}_{2}$O${}_{8}$	Immm [260]	?	APBs [260]	?
Ca${}_{1-x}$Sr${}_{x}$Al${}_{2}$Si${}_{2}$O${}_{8}$ (x>0.85)	I$\frac{2}{c}$ [261]	?	?	?
Ca${}_{1-x}$Sr${}_{x}$Al${}_{2}$Si${}_{2}$O${}_{8}$ (x= 0.60–0.85)	I$\frac{2}{c}$ [262,263]	O disorder [264]	APBs [264]	albite twins [264]
Ca${}_{1-x}$Sr${}_{x}$Al${}_{2}$Si${}_{2}$O${}_{8}$ (x<0.5)	I$\bar{1}$	Al-Si disorder [256]	?	?
Ca${}_{2}$MgSi${}_{2}$O${}_{7}$	P$\bar{4}2_{1}m$ [265] (>355 K)	modulation [223]	micro [225,266]	?
Ca${}_{2}$Al${}_{2}$SiO${}_{7}$	P$\bar{4}2_{1}m$	Al-Si order@T${}^{2}$, Al partly order@T${}^{1}$	?	?
Ca${}_{1-x}$Sr${}_{x}$MgSi${}_{2}$O${}_{7}$ (x<0.32)	P$\bar{4}2_{1}m$ 350–295 K	‘Titan’ modulation, Sr order [225,266]	micro [225,266]	?
Ca${}_{2}$(Al${}_{x}$Mg${}_{1-x}$)-(Al${}_{x}$Si${}_{2-x}$)O${}_{7}$	?	Al, Si order [228]		twins [228]
CaTiO${}_{3}$	?	Ti displacement	?	twins [267,268]
BaTiO${}_{3}$	Pm$\bar{3}$m	Ti displacement	ferroelectric ferroelastic	twins [269]
LiNbO${}_{3}$	R$\bar{3}$c (>1470 K)	Li [180] vacancies	?	?
Sr${}_{2}$SnO${}_{4}$	Bmab [270] (400–600 K) I$\frac{4}{m}$mm [270] (>600 K)	vacancies	?	?
α-CaZr(PO${}_{4}$)${}_{2}$	Pnma [193] (>1000 ${}^{\circ }$C)	?	?	?
BaSi${}_{2}$O${}_{2}$N${}_{2}$	?	disorder [241]	domains [192,241]	twins [271]
SrSi${}_{2}$O${}_{2}$N${}_{2}$	?	?	domains [271]	?
ZrO${}_{2}$	P$\frac{4_{2}}{n}$mc [272] (>1500 K)	?	?	?

**Notes**: HT Phase ${}^{⋄}$: high temperature phase; Atomic Order ${}^{♡}$: q is the modulation vector; Domains ${}^{▹}$: APBs = anti-phase boundaries; ? = data unavailable.

**Figure A1:**
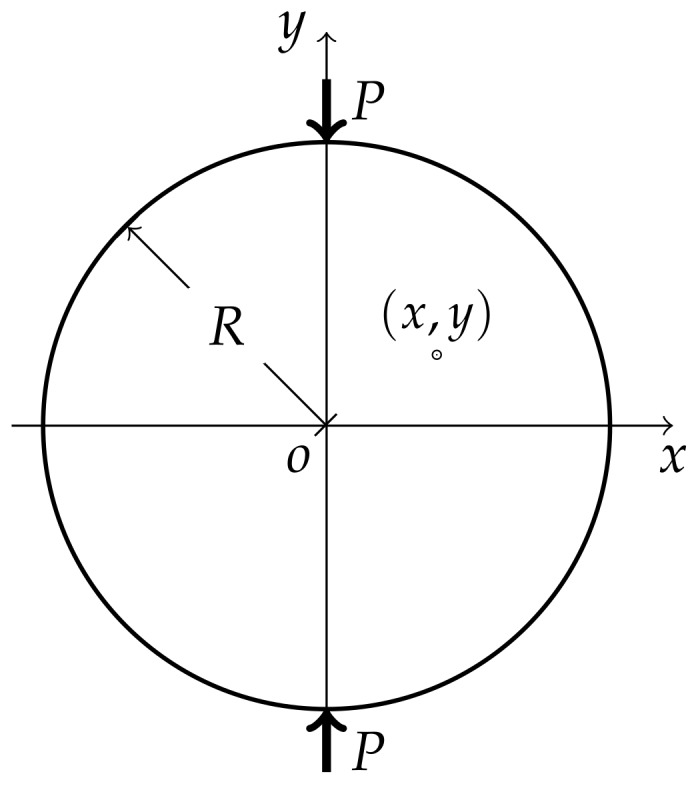
Schematic view of a disc with diameter 2R and thickness *l* subjected to a diametrical load *P*.

**Figure A2:**
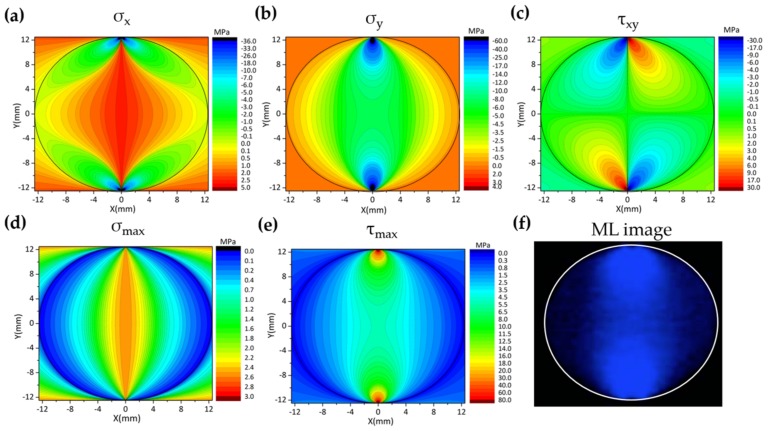
The distribution of components of stress tensor $\sigma_{x}$ (**a**); $\sigma_{y}$ (**b**); and $\tau_{xy}$ (**c**) respectively and the corresponding distribution of maximum tensile stress $\sigma_{max}$ (**d**) and maximum shear stress $\tau_{max}$ (**e**). An optical image of ML in CaYAl${}_{3}$O${}_{7}$: Eu phosphor (**f**) (Reproduced from Ref [165]. Copyright 2008, The Electrochemical Society.) in a composite disc (diameter 2R=25 mm, thickness l=15 mm) under load P=1000 N. Solid circles exhibit the boundary of the disc.

## Appendix A. Stress Distribution of a Disc Under a Pair of Diametrical Compressive Load

The experimental set-up for sensing stress distribution via ML (Figure 3a) can be summarized as a pair of equal load *P* applied diametrically to a disc with diameter 2R and thickness *l* (Figure A1).

The analytical solutions of tensile stress $\sigma_{x}$, $\sigma_{y}$ and shear stress $\tau_{xy}$ in the neighbourhood of a point (x,y) in the surface plane can be found as [343]

(A1) $$\sigma_{x}=-\frac{2P}{\pi l}\left\{\frac{\left(R-y\right)x^{2}}{{\left[{\left(R-y\right)}^{2}+x^{2}\right]}^{2}}+\frac{\left(R+y\right)x^{2}}{{\left[{\left(R+y\right)}^{2}+x^{2}\right]}^{2}}-\frac{1}{2R}\right\}$$

(A2) $$\sigma_{y}=-\frac{2P}{\pi l}\left\{\frac{{\left(R-y\right)}^{3}}{{\left[{\left(R-y\right)}^{2}+x^{2}\right]}^{2}}+\frac{{\left(R+y\right)}^{3}}{{\left[{\left(R+y\right)}^{2}+x^{2}\right]}^{2}}-\frac{1}{2R}\right\}$$

(A3) $$\tau_{xy}=\frac{2P}{\pi l}\left\{\frac{{\left(R-y\right)}^{2}x}{{\left[{\left(R-y\right)}^{2}+x^{2}\right]}^{2}}-\frac{{\left(R+y\right)}^{2}x}{{\left[{\left(R+y\right)}^{2}+x^{2}\right]}^{2}}\right\}$$

The maximum shear stress $\tau_{max}$ and the maximum tensile stress $\sigma_{mx}$ are calculated by:

(A4) $$\tau_{max}=\sqrt{{\left(\sigma_{x}-\sigma_{y}\right)}^{2}/4+\tau_{xy}^{2}}$$

(A5) $$\sigma_{max}=\left(\sigma_{x}+\sigma_{y}\right)/2+\tau_{max}$$

For a typical ML experiment, R= 12.5 mm, l= 10 mm, and P= 1000 N, the components of stress tensor, the maximum tensile tress and the maximum shear stress are calculated and visualized in Figure A2. It is clear that the distribution of ML intensity agrees well with $\tau_{max}$, which is directly related to the isochromatic fringes in photoelasticity.

## Appendix B. Influence of Point Group on the Components of Piezoelectric Tensor and Piezo-Optical Tensor

Neumann’s principle places symmetry restrictions on the tensors of physical properties. Furthermore, some symmetry of the interested physical quantity itself also influence the relations of tensor components. Her, the piezoelectric tensor is shown how it is influenced by the symmetry of static stress and the point group of a crystal. The piezoelectricity effect relate electric polarization $P_{i}$ to stress $\sigma_{jk}$ by the relation:

(A6) $$P_{i}=d_{ijk}\sigma_{jk}$$

where $d_{ijk}$ is the third rank piezoelectric tensor. The symmetry of static stress tensor $\sigma_{jk}$ reduced the number of independent components from 27 to 18. For a specific point group, e.g., 4 (rotation axis along $x_{3}$), applying Neumann’s principle via the direct inspection method results new relations between these components. Rotation 4 induces the transform of axis as $x_{1}\to x_{2},x_{2}\to x_{1},x_{3}\to x_{3}$, thus piezoelectric constants transform as follows:

$$d_{111}\to d_{222}\to -d_{222},d_{11}=d_{22}=0;$$

$$d_{122}\to d_{211}\to -d_{122},d_{12}=d_{21}=0;$$

$$d_{133}\to d_{233}\to -d_{133},d_{13}=d_{23}=0;$$

$$d_{123}\to -d_{213}\to d_{123},d_{14}=-d_{25};$$

$$d_{131}\to d_{232}\to d_{131},d_{15}=d_{24};$$

$$d_{112}\to -d_{221}\to -d_{112},d_{16}=-d_{25}=0;$$

$$d_{311}\to d_{322}\to d_{311},d_{31}=d_{32};$$

$$d_{323}\to -d_{313}\to -d_{311},d_{34}=-d_{35}=0;$$

$$d_{312}\to -d_{321},d_{36}=0;$$

$$d_{333}\to d_{333}.$$

Here, the Voigt notation was applied: 11→1,22→2,33→3,23→4,13→5,12→6 . Thus, there are four independent piezoelectric components, namely $d_{14}=-d_{25},d_{15}=d_{24},d_{31}=d_{32},d_{33}$, while other components are zero. In any centrosymmetric crystals, an inversion centre leads to $d_{ijk}=0$ because the vector basis transform requires $d_{ijk}^{\prime }=-d_{ijk}$, while Neumann’s principle asks for $d_{ijk}^{\prime }=d_{ijk}$.

The photoelastic effect is the phenomenon that application of stress changes the refractive index of a crystal. It can be represented as:

(A7) $$▵B_{ij}=\pi_{ijkl}\sigma_{kl}$$

since the refractive index is specified by the indicatrix which is an ellipsoid whose coefficients are the components of relative dielectric impermeability tensor $B_{ij}$ (a second rank tensor). $\pi_{ijkl}$ is the piezo-optical coefficients, a fourth-rank tensor and the number of components is 81. The symmetry of both $B_{ij}$ and $\sigma_{kl}$ leads to $\pi_{mn}{=\{}_{2\pi_{ijkl},n=4,5,6}^{\pi_{ijkl},n=1,2,3}$ which satisfy $▵B_{m}=\pi_{mn}\sigma_{n}\left(m,n=1,2,\cdots ,6\right)$, but generally $\pi_{mn}\neq \pi_{nm}$. As an example, the point group 23 (cubic system) will lead to relations in $\pi^{\prime }s$ by direct inspection method. A triad axis along <111> changes the suffixes as 1→2,2→3,3→1 ,which cause the suffix of tensor transform as (in two-suffix form) 1→2,2→3,3→1,4→5,5→6,6→4. Hence, the relations between components are [45]

$$\pi_{11}=\pi_{22}=\pi_{33};\pi_{12}=\pi_{23}=\pi_{31};$$

$$\pi_{13}=\pi_{21}=\pi_{32};\pi_{44}=\pi_{55}=\pi_{66}.$$

## Appendix C. An Estimation of Piezoelectric Field in ZnS Crystallite

ZnS (wurtzite) is a piezoelectric semiconductor, with space group $P6_{3}mc$. The piezoelectric tensor is [202]

$$d=\left[\begin{array}{cccccc} 0 & 0 & 0 & 0 & -2.8 & 0\\ 0 & 0 & 3.2 & -2.8 & 0 & 0\\ -1.1 & -1.1 & 0 & 0 & 0 & 0 \end{array}\right]\mathrm{pC}/N$$

and the electric field due to shear stress is

(A8) $$E_{i}=\frac{d_{15}\sigma_{i\neq j}}{\varepsilon_{0}\left(1+\chi \right)}$$

in which $d_{15}=-2.8$ pC/N, the permittivity of vacuum $\varepsilon_{0}=8.85$ pC/(Vm), and the relative dielectric constant of ZnS (1+χ)=9.6. Therefore,

(A9) $$E_{i}\left(V/\mathrm{cm}\right)=-3.2\times 10^{-4}\;\sigma \left(\mathrm{Pa}\right)$$

which is also applicable to ZnS doped with divalent cations. The critical electric field for electroluminescence is around $10^{5}$ V/cm [304], which is ∼100 times larger than the piezoelectric field at a stress of 100 MPa.
